# A statistical measure for the skewness of X chromosome inactivation for quantitative traits and its application to the MCTFR data

**DOI:** 10.1186/s12863-021-00978-z

**Published:** 2021-07-02

**Authors:** Bao-Hui Li, Wen-Yi Yu, Ji-Yuan Zhou

**Affiliations:** 1 Department of Biostatistics, State Key Laboratory of Organ Failure Research, Ministry of Education, and Guangdong Provincial Key Laboratory of Tropical Disease Research, School of Public Health, Southern Medical University, No. 1023, South Shatai Road, Baiyun District, Guangzhou, 510515 Guangdong China; 2 Guangdong-Hong Kong-Macao Joint Laboratory for Contaminants Exposure and Health, Guangzhou, 510006 China

**Keywords:** X chromosome inactivation, Skewness, Quantitative trait, Variance heterogeneity, Minnesota Center for Twin and Family Research data

## Abstract

**Background:**

X chromosome inactivation (XCI) is that one of two chromosomes in mammalian females is silenced during early development of embryos. There has been a statistical measure for the degree of the skewness of XCI for qualitative traits. However, no method is available for such task at quantitative trait loci.

**Results:**

In this article, we extend the existing statistical measure for the skewness of XCI for qualitative traits, and the likelihood ratio, Fieller’s and delta methods for constructing the corresponding confidence intervals, and make them accommodate quantitative traits. The proposed measure is a ratio of two linear regression coefficients when association exists. Noting that XCI may cause variance heterogeneity of the traits across different genotypes in females, we obtain the point estimate and confidence intervals of the measure by incorporating such information. The hypothesis testing of the proposed methods is also investigated. We conduct extensive simulation studies to assess the performance of the proposed methods. Simulation results demonstrate that the median of the point estimates of the measure is very close to the pre-specified true value. The likelihood ratio and Fieller’s methods control the size well, and have the similar test power and accurate coverage probability, which perform better than the delta method. So far, we are not aware of any association study for the X-chromosomal loci in the Minnesota Center for Twin and Family Research data. So, we apply our proposed methods to these data for their practical use and find that only the rs792959 locus, which is simultaneously associated with the illicit drug composite score and behavioral disinhibition composite score, may undergo XCI skewing. However, this needs to be confirmed by molecular genetics.

**Conclusions:**

We recommend the Fieller’s method in practical use because it is a non-iterative procedure and has the similar performance to the likelihood ratio method.

**Supplementary Information:**

The online version contains supplementary material available at 10.1186/s12863-021-00978-z.

## Background

In genome-wide association study (GWAS), many human diseases have been found to be associated with X-chromosomal genes, such as autoimmune diseases [1, 2], asthma [3], Duchenne muscular dystrophy [4, 5], adrenoleukodystrophy [6], Wiskott-Aldrich syndrome [7] and some cancers [8–12]. However, development of methods for identifying association with genetic variants on X chromosome still lags behind that on autosomes due to the unique inheritance pattern of X chromosome [13]. The number of X chromosomes is different between males and females in mammals. There are two copies of X chromosome in mammalian females, one of which is paternal and the other is maternal, while mammalian males have only one maternal X chromosome. To compensate for this X chromosome dosage difference between sexes, one of two chromosomes in females is silenced during the early development of embryos, which is called X chromosome inactivation (XCI) [14–18]. Random XCI (XCI-R) is a process that either the paternal or the maternal allele at an X-chromosomal locus is randomly chosen to be silenced in all cells, which is common in most females [19]. However, skewed XCI (XCI-S) is also observed in a proportion of females, which is a non-random process and is defined as the observation of inactivation of the same allele in more than 75% cells [9, 20–23]. In addition, not all of the X-linked genes undergo XCI and the pseudo-autosomal region on both sex chromosomes does not require dosage compensation. In humans, over 15% X-linked genes have been shown to escape from XCI (XCI-E) [24, 25].

In population genetics, there has been an increasing interest in the incorporation of the information on XCI into association analysis for qualitative traits [26–30] and quantitative traits [31–34], which may greatly improve the test power. For qualitative traits, Clayton [26] first took account of XCI in detecting the association between X-chromosomal markers and diseases by regarding males as homozygous females. However, the Clayton’s method only considers the XCI-R pattern and does not incorporate the XCI-S and XCI-E patterns. So, Wang et al. [27] developed a resampling-based approach for case-control data simultaneously combining the information on three XCI patterns (XCI-R, XCI-S and XCI-E) by coding three genotypes in females as 0, *γ* and 2, where *γ* is an unknown parameter, takes possible values between 0 and 2, and can be used to measure the degree of the skewness of XCI. For X-linked quantitative trait loci (QTL), Zhang et al. [31] proposed a family-based association test, where the quantitative trait under study is required to follow a normal distribution. Although the involved variances of the trait value for males and females are assumed to be different, those for three genotypes in females are fixed to be the same. However, according to Ma et al. [32], XCI may lead to variance heterogeneity of the traits across different genotypes in females and the variance of the trait in heterozygous females is generally higher than that in homozygous females. So, based on only unrelated females, Ma et al. [32] suggested a test for X-linked association via inflated variance in heterozygous females, a weighted test for X-linked association which considers different variances, and the combined test of these two tests using the Stouffer’s Z-score method. Gao et al. [33] further developed the XWAS software toolset to facilitate GWAS on X chromosome, which includes the three test statistics proposed by Ma et al. [32]. Deng et al. [34] put forward a sex-specific Levene’s test, and a generalized Levene’s test based on a two-stage regression model accounting for sex-specific mean and variance effects, to test for association. The original Levene’s test is robust to certain types of non-normal distribution, particularly when data are non-normal but symmetric [34], while the generalized Levene’s test may not. It should be noted that the above methods for QTL only incorporate the XCI-R and XCI-E patterns and do not consider the XCI-S pattern. On the other hand, Wang et al. [35] has recently proposed a statistical measure available for the degree of XCI skewing for case-control data and developed three methods (likelihood ratio (LR), Fieller’s and delta) to construct the corresponding confidence intervals (CIs). However, they are only applicable to qualitative traits and are not suitable for quantitative traits.

Therefore, in this article, we first extend the existing statistical measure for the degree of XCI skewing (i.e., *γ*) for qualitative traits [35] and make it accommodate quantitative traits. It is shown that the proposed *γ* is a ratio of two linear regression coefficients in the presence of association between the traits under study and the genotypes. We estimate the linear regression coefficients by incorporating the information on the variance heterogeneity across different genotypes in females and then obtain the point estimate of *γ*. Then, we extend the existing LR, Fieller’s and delta methods for constructing the CIs of *γ* and make them suitable for quantitative traits. The simulation studies under various simulation settings are conducted to assess the performance of the proposed methods. We also apply the proposed methods to the Minnesota Center for Twin and Family Research (MCTFR) data for their practical use. Note that so far, we are not aware of any association study for the X-chromosomal markers in the MCTFR data, although there have been some previous association studies which only focused on autosomal markers [36–43].

## Results

### Sizes and powers

The empirical type I error rates of the corresponding tests for the proposed LR, Fieller’s and delta methods based on the sample size *n* = 1,000 and 2,000 are respectively given in Tables 1 and 2, where the additive effect size *a* = 0.1 and 0.3, the allele frequency *p* = 0.1 and 0.3, and the inbreeding coefficient *ρ* = 0. Under all the situations considered, the sizes of the proposed LR and Fieller’s methods stay close to the pre-specified nominal level of 5%, irrespective of the values of *n*, *a* and *p*, which verifies their validity. However, the delta method has the inflated or conservative type I error rates in most scenarios. Additional file 1: Tables S1 and S2 show the sizes for the proposed LR, Fieller’s and delta methods with *ρ* = 0.05 based on the sample size *n* = 1,000 and 2,000, respectively, which are similar to those in Tables 1 and 2. This demonstrates that the Hardy-Weinberg disequilibrium almost has no effect on the sizes.

**Table 1:** Estimated sizes (in %) for testing *H*_0_ : *γ* = *γ*_0_ for the LR, Fieller’s and delta methods with *a* = 0.1 and 0.3, *p* = 0.1 and 0.3, *n* = 1,000 and *ρ* = 0 based on 10,000 replicates and 5% significance level

*a*	*p*	*γ*_0_	LR	Fieller	Delta
0.1	0.1	0	4.96	4.87	0.24
0.1	0.1	0.5	5.11	5.00	6.24
0.1	0.1	1	4.89	4.80	10.27
0.1	0.1	1.5	5.30	5.23	11.13
0.1	0.1	2	4.96	4.98	11.16
0.1	0.3	0	4.92	4.90	2.86
0.1	0.3	0.5	5.29	5.24	3.13
0.1	0.3	1	4.71	4.74	3.77
0.1	0.3	1.5	5.19	5.02	5.15
0.1	0.3	2	5.02	4.90	5.47
0.3	0.1	0	5.10	5.07	0.26
0.3	0.1	0.5	5.05	5.05	5.73
0.3	0.1	1	5.04	5.00	10.33
0.3	0.1	1.5	5.09	5.07	11.51
0.3	0.1	2	5.13	5.19	11.34
0.3	0.3	0	4.94	4.85	2.79
0.3	0.3	0.5	4.91	4.92	2.90
0.3	0.3	1	5.24	5.23	4.46
0.3	0.3	1.5	5.07	5.05	5.05
0.3	0.3	2	4.82	4.89	5.12

**Table 2:** Estimated sizes (in %) for testing *H*_0_ : *γ* = *γ*_0_ for the LR, Fieller’s and delta methods with *a* = 0.1 and 0.3, *p* = 0.1 and 0.3, *n* = 2,000 and *ρ* = 0 based on 10,000 replicates and 5% significance level

*a*	*p*	*γ*_0_	LR	Fieller	Delta
0.1	0.1	0	5.22	5.10	0.64
0.1	0.1	0.5	5.06	4.99	6.43
0.1	0.1	1	4.93	4.97	8.63
0.1	0.1	1.5	5.05	5.03	9.28
0.1	0.1	2	4.93	4.92	9.50
0.1	0.3	0	4.85	4.95	2.88
0.1	0.3	0.5	5.17	5.14	4.35
0.1	0.3	1	4.82	4.80	4.14
0.1	0.3	1.5	5.34	5.30	4.50
0.1	0.3	2	5.10	5.12	4.69
0.3	0.1	0	5.30	5.21	0.57
0.3	0.1	0.5	5.21	5.31	6.27
0.3	0.1	1	5.11	5.05	8.44
0.3	0.1	1.5	4.97	4.91	8.84
0.3	0.1	2	4.83	4.83	9.20
0.3	0.3	0	5.15	5.18	2.97
0.3	0.3	0.5	4.84	4.89	3.79
0.3	0.3	1	5.02	5.01	4.34
0.3	0.3	1.5	5.24	5.22	4.52
0.3	0.3	2	5.20	5.21	4.81

**Fig. 1:**
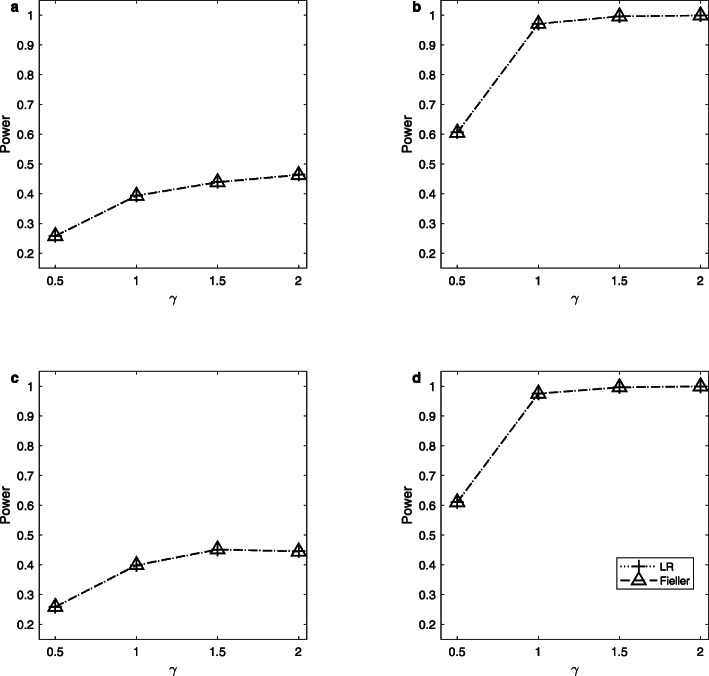
Estimated powers for the LR and Fieller’s methods against *γ*. The simulation is based on 10,000 replicates and 5% significance level with *n* = 1,000, *ρ* = 0 and *γ*_0_ = 0. **a** *a* = 0.1, *p* = 0.1; **b** *a* = 0.1, *p* = 0.3; **c** *a* = 0.3, *p* = 0.1; **d** *a* = 0.3, *p* = 0.3

**Fig. 2:**
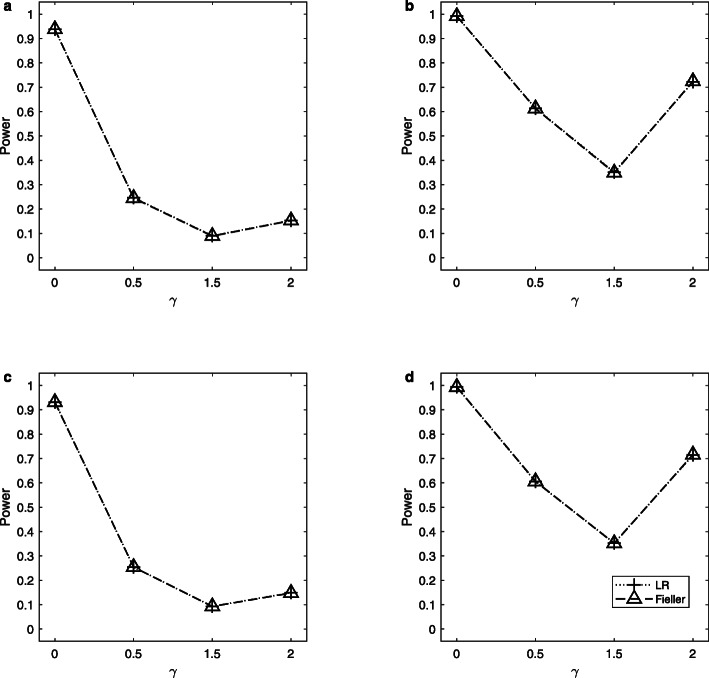
Estimated powers for the LR and Fieller’s methods against *γ*. The simulation is based on 10,000 replicates and 5% significance level with *n* = 1,000, *ρ* = 0 and *γ*_0_ = 1. **a** *a* = 0.1, *p* = 0.1; **b** *a* = 0.1, *p* = 0.3; **c** *a* = 0.3, *p* = 0.1; **d** *a* = 0.3, *p* = 0.3

**Fig. 3:**
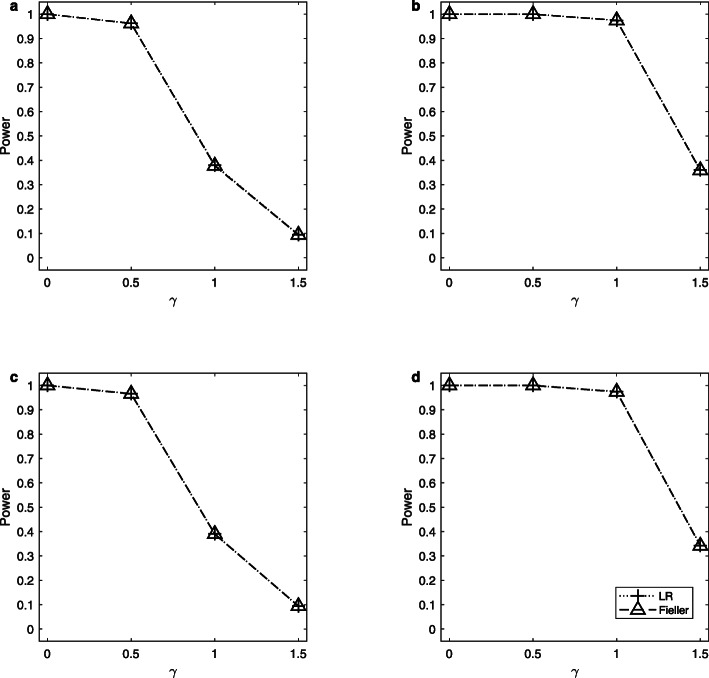
Estimated powers for the LR and Fieller’s methods against *γ*. The simulation is based on 10,000 replicates and 5% significance level with *n* = 1,000, *ρ* = 0 and *γ*_0_ = 2. **a** *a* = 0.1, *p* = 0.1; **b** *a* = 0.1, *p* = 0.3; **c** *a* = 0.3, *p* = 0.1; **d** *a* = 0.3, *p* = 0.3

**Fig. 4:**
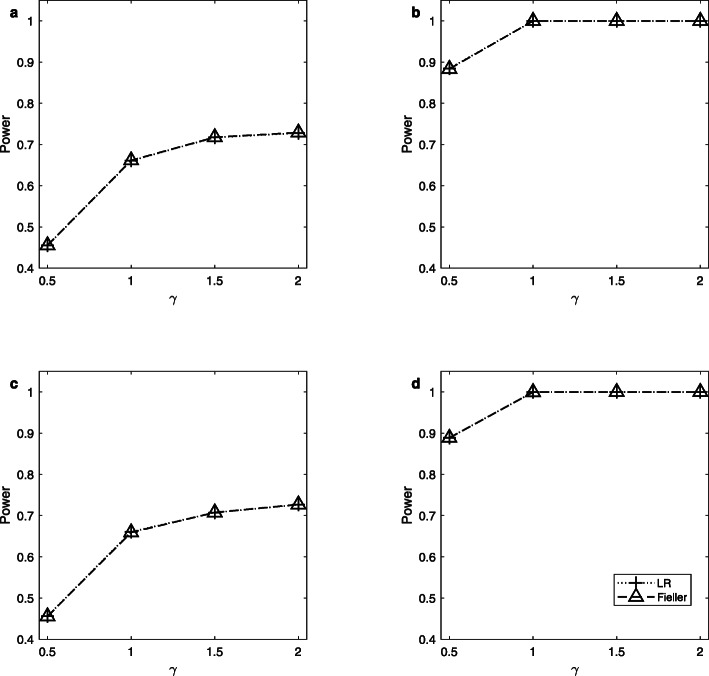
Estimated powers for the LR and Fieller’s methods against *γ*. The simulation is based on 10,000 replicates and 5% significance level with *n* = 2,000, *ρ* = 0 and *γ*_0_ = 0. **a** *a* = 0.1, *p* = 0.1; **b** *a* = 0.1, *p* = 0.3; **c** *a* = 0.3, *p* = 0.1; **d** *a* = 0.3, *p* = 0.3

**Fig. 5:**
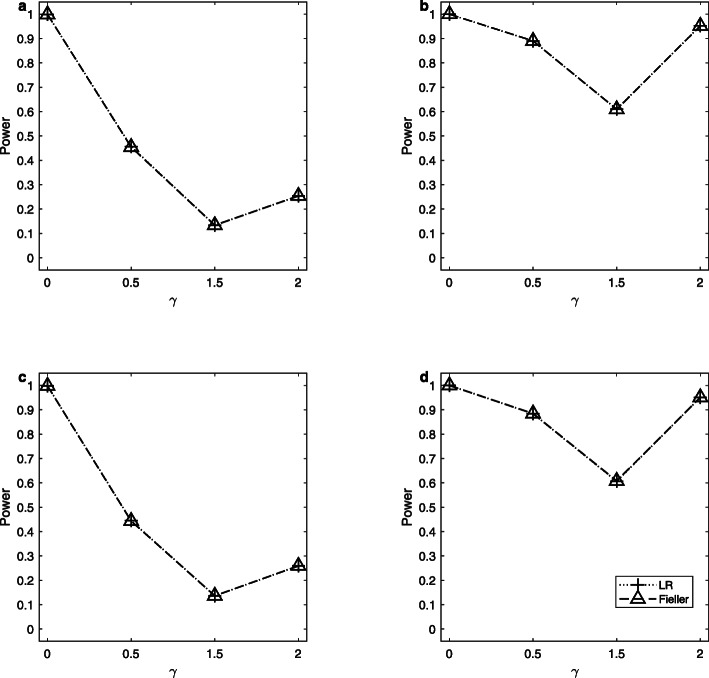
Estimated powers for the LR and Fieller’s methods against *γ*. The simulation is based on 10,000 replicates and 5% significance level with *n* = 2,000, *ρ* = 0 and *γ*_0_ = 1. **a** *a* = 0.1, *p* = 0.1; **b** *a* = 0.1, *p* = 0.3; **c** *a* = 0.3, *p* = 0.1; **d** *a* = 0.3, *p* = 0.3

**Fig. 6:**
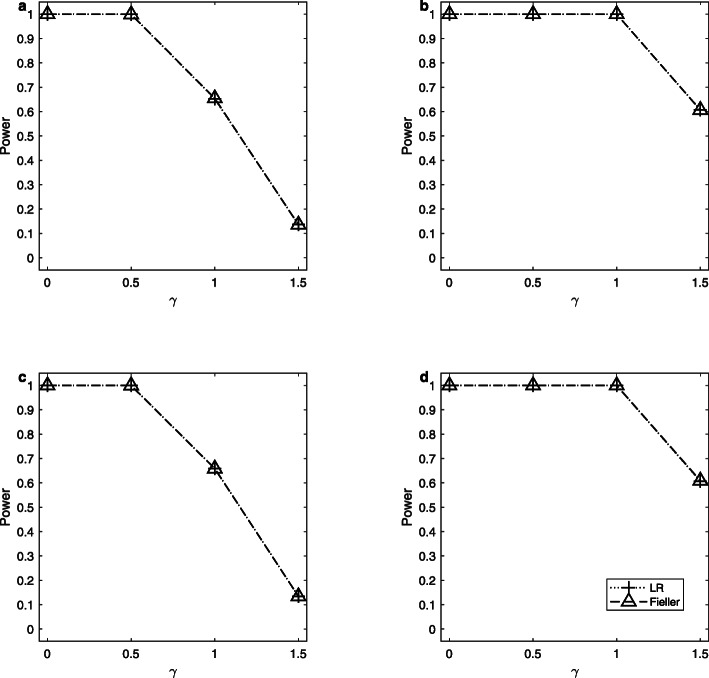
Estimated powers for the LR and Fieller’s methods against *γ*. The simulation is based on 10,000 replicates and 5% significance level with *n* = 2,000, *ρ* = 0 and *γ*_0_ = 2. **a** *a* = 0.1, *p* = 0.1; **b** *a* = 0.1, *p* = 0.3; **c** *a* = 0.3, *p* = 0.1; **d** *a* = 0.3, *p* = 0.3

Note that the delta method does not control the sizes well. So, we only simulate the powers of the LR and Fieller’s methods. Figures 1, 2 and 3 display the estimated powers for the LR and Fieller’s methods against *γ* (*γ* ≠ *γ*_0_) with *a* = 0.1 and 0.3, *p* = 0.1 and 0.3, *n* = 1,000, and *ρ* = 0 when *γ*_0_ = 0, 1 and 2, respectively. Figures 4, 5 and 6 plot the corresponding estimated powers with *a* = 0.1 and 0.3, *p* = 0.1 and 0.3, *n* = 2,000, and *ρ* = 0 when *γ*_0_ = 0, 1 and 2, respectively. The other power results are shown in Additional file 1: Figures S1-S14. It can be seen from these figures that the power of the LR method is almost the same as that of the Fieller’s method. The powers of the LR and Fieller’s methods gradually but asymmetrically become larger with ∣*γ* − *γ*_0_∣ increasing. When other parameters are unchanged, the powers with *p* = 0.3 are bigger than those with *p* = 0.1 (e.g., Fig. 1b vs. Fig. 1a, Fig. 1d vs. Fig. 1c). However, note that in ${\sigma}_1^2=\theta \left(1-\theta \right){a}^2+1.1$, *θ*(1 − *θ*)*a*^2^ attains its maximum 0.25*a*^2^ when *θ* = 0.5 (i.e., *γ* = 1). The corresponding values of ${\sigma}_1^2$ for *a* = 0.1 and 0.3 are 1.1025 and 1.1225, respectively, which are not so different from each other. Furthermore, when *γ* = 0 or 2, *θ*(1 − *θ*)*a*^2^ = 0, which is not related to the value of *a*. So, the powers with *a* = 0.1 and those with *a* = 0.3 are close to each other (e.g., Fig. 1a vs. Fig. 1c, Fig. 1b vs. Fig. 1d). When the sample size *n* is changed from 1,000 to 2,000, the LR and Fieller’s methods are more powerful (e.g., Fig. 4 vs. Fig. 1). Finally, we find that the Hardy-Weinberg disequilibrium has little influence on the power results, e.g., by comparing Fig. 1 (*ρ* = 0) with Additional file 1: Figure S3 (*ρ* = 0.05).

### Median of point estimate and statistical properties of confidence intervals

Tables 3 and 4 show the estimated median of the point estimates of *γ*, CP, ML, MR, ML/(ML + MR), DP and EP of the two-sided 95% CIs of *γ* for the LR, Fieller’s and delta methods against *γ*, with *a* = 0.1 and 0.3, *p* = 0.1 and 0.3, and *ρ* = 0 based on 10,000 replicates for *n* = 1,000 and 2,000, respectively. From these two tables, we find that in all the cases considered, the median of $\hat{\gamma}$ maintains very close to the true value of *γ*. As for the CI, the LR and Fieller’s methods have similar performance in the CP and the CPs of both methods are controlled around 95%, regardless of the values of *a*, *p*, *γ* and *n*. However, the CP of the delta method is underestimated or overestimated in most of the considered situations. The values of the ML/(ML + MR) for the LR and Fieller’s methods generally stay close to 0.5, except for the cases of *p* = 0.1 and *n* = 1,000, and the situations of *γ* = 0 and 2, while the ML/(ML + MR) of the delta method always gets far way from 0.5. This indicates that the LR and Fieller’s methods achieve more balance between ML and MR than the delta method. The LR and Fieller’s methods have comparable performance in the DP and EP. The values of the DP of both methods are zero or close to zero, except for *p* = 0.1 and *γ* = 0, which is indicative of few discontinuous CIs to occur. However, the EP results of the LR and Fieller’s methods show that there still exist a few CIs which are empty sets or reduced to be a point. On the other hand, the DP and EP of the delta method are zero for all the simulation settings. This is because the CI based on the delta method is always bounded and is a continuous interval. The ML/(ML + MR), DP and EP of the LR and Fieller’s methods appear not to be greatly affected by the values of *a* (0.1 or 0.3), while the LR and Fieller’s methods perform worse in the ML/(ML + MR) and the DP when *p* = 0.1, compared to those with *p* = 0.3. When the sample size increases from 1,000 (Table 3) to 2,000 (Table 4), the LR and Fieller’s methods have more balance of two tail errors and the values of the DP for *p* = 0.1 and *γ* = 0 are less. When *γ*= 0.5, 1 and 1.5, the values of the EP of both methods with *p* = 0.3 are less than those with *p* = 0.1, and the LR and Fieller’s methods with *n* = 2,000 have smaller EP values than *n* = 1,000. However, when *γ*= 0 and 2, the corresponding values of the EP with *p* = 0.3 are a little larger than those with *p* = 0.1 and the values of the EP with *n* = 2,000 are a little bigger than those with *n* = 1,000. This may be because *γ*= 0 and 2 are the endpoints of the interval [0, 2], which are the extreme cases. The corresponding results of the median of $\hat{\gamma}$, CP, ML, MR, ML/(ML + MR), DP and EP of the 95% CIs of *γ* for the LR, Fieller’s and delta methods with *ρ* = 0.05 for *n* = 1,000 and 2,000 are given in Additional file 1: Tables S3 and S4, respectively. By comparing Table 3 with Additional file 1: Table S3 (or comparing Table 4 with Additional file 1: Table S4), we can see that the results in both tables are similar to each other, which means that the Hardy-Weinberg disequilibrium has no great effect on the point estimation and the interval estimation of *γ*.

**Table 3:** Estimated median of the point estimates of *γ*, CP (in %), ML (in %), MR (in %), Ratio (ML/(ML + MR)), DP (in %) and EP (in %) of two-sided 95% CIs of *γ* for the LR, Fieller’s and delta methods against *γ*, with *a* = 0.1 and 0.3, *p* = 0.1 and 0.3, *n* = 1,000 and *ρ* = 0 based on 10,000 replicates

				LR						Fieller						Delta^a^			
*a*	*p*	*γ*	Median	CP	ML	MR	Ratio	DP	EP	CP	ML	MR	Ratio	DP	EP	CP	ML	MR	Ratio
0.1	0.1	0	0.01	95.04	2.53	0.00	1.00	2.02	1.58	95.13	2.48	0.00	1.00	1.97	1.51	99.76	0.24	0.00	1.00
0.1	0.1	0.5	0.47	94.89	1.57	2.44	0.39	0.20	0.06	95.00	1.54	2.36	0.39	0.20	0.06	93.76	0.00	6.24	0.00
0.1	0.1	1	0.95	95.11	0.91	2.59	0.26	0.03	0.21	95.20	0.87	2.58	0.25	0.02	0.19	89.73	0.00	10.27	0.00
0.1	0.1	1.5	1.42	94.70	0.66	2.51	0.21	0.00	0.83	94.77	0.67	2.45	0.21	0.00	0.83	88.87	0.00	11.13	0.00
0.1	0.1	2	1.89	95.04	0.00	2.55	0.00	0.00	2.40	95.02	0.00	2.56	0.00	0.00	2.42	88.84	0.00	11.16	0.00
0.1	0.3	0	0.00	95.08	2.57	0.00	1.00	0.00	2.35	95.10	2.53	0.00	1.00	0.00	2.37	97.14	2.86	0.00	1.00
0.1	0.3	0.5	0.50	94.71	2.70	2.58	0.51	0.00	0.01	94.76	2.64	2.59	0.50	0.00	0.01	96.87	1.10	2.03	0.35
0.1	0.3	1	1.00	95.29	2.43	2.27	0.52	0.00	0.00	95.26	2.45	2.29	0.52	0.00	0.00	96.23	0.12	3.65	0.03
0.1	0.3	1.5	1.50	94.81	2.64	2.50	0.51	0.00	0.05	94.98	2.52	2.45	0.51	0.00	0.05	94.85	0.00	5.15	0.00
0.1	0.3	2	2.00	94.98	0.00	2.51	0.00	0.00	2.51	95.10	0.00	2.43	0.00	0.00	2.47	94.53	0.00	5.47	0.00
0.3	0.1	0	0.00	94.90	2.52	0.00	1.00	2.01	1.73	94.93	2.56	0.00	1.00	2.08	1.69	99.74	0.26	0.00	1.00
0.3	0.1	0.5	0.47	94.95	1.49	2.53	0.37	0.15	0.06	94.95	1.49	2.54	0.37	0.16	0.06	94.27	0.00	5.73	0.00
0.3	0.1	1	0.95	94.96	0.79	2.73	0.22	0.07	0.23	95.00	0.82	2.65	0.24	0.07	0.19	89.67	0.00	10.33	0.00
0.3	0.1	1.5	1.39	94.91	0.67	2.69	0.20	0.00	0.85	94.93	0.64	2.67	0.19	0.00	0.81	88.49	0.00	11.51	0.00
0.3	0.1	2	1.92	94.87	0.00	2.57	0.00	0.00	2.56	94.81	0.00	2.56	0.00	0.00	2.63	88.66	0.00	11.34	0.00
0.3	0.3	0	0.00	95.06	2.43	0.00	1.00	0.00	2.51	95.15	2.37	0.00	1.00	0.00	2.48	97.21	2.79	0.00	1.00
0.3	0.3	0.5	0.50	95.09	2.50	2.39	0.51	0.00	0.00	95.08	2.50	2.42	0.51	0.00	0.00	97.10	0.99	1.91	0.34
0.3	0.3	1	1.00	94.76	2.56	2.68	0.49	0.00	0.00	94.77	2.56	2.67	0.49	0.00	0.00	95.54	0.12	4.34	0.03
0.3	0.3	1.5	1.50	94.93	2.39	2.55	0.48	0.00	0.10	94.95	2.35	2.59	0.48	0.00	0.11	94.95	0.00	5.05	0.00
0.3	0.3	2	2.00	95.18	0.00	2.20	0.00	0.00	2.61	95.11	0.00	2.21	0.00	0.00	2.68	94.88	0.00	5.12	0.00

^a^ DP and EP of the delta method are zero

**Table 4:** Estimated median of the point estimates of *γ*, CP (in %), ML (in %), MR (in %), Ratio (ML/(ML + MR)), DP (in %) and EP (in %) of two-sided 95% CIs of *γ* for the LR, Fieller’s and delta methods against *γ*, with *a* = 0.1 and 0.3, *p* = 0.1 and 0.3, *n* = 2,000 and *ρ* = 0 based on 10,000 replicates

				LR						Fieller						Delta^a^			
*a*	*p*	*γ*	Median	CP	ML	MR	Ratio	DP	EP	CP	ML	MR	Ratio	DP	EP	CP	ML	MR	Ratio
0.1	0.1	0	0.00	94.78	2.70	0.00	1.00	1.22	2.16	94.90	2.60	0.00	1.00	1.12	2.13	99.36	0.64	0.00	1.00
0.1	0.1	0.5	0.49	94.94	2.17	2.68	0.45	0.00	0.00	95.01	2.12	2.69	0.44	0.00	0.00	93.57	0.00	6.43	0.00
0.1	0.1	1	1.00	95.07	2.12	2.40	0.47	0.00	0.05	95.03	2.13	2.42	0.47	0.00	0.07	91.37	0.00	8.63	0.00
0.1	0.1	1.5	1.48	94.95	1.89	2.56	0.42	0.00	0.44	94.97	1.91	2.52	0.43	0.00	0.44	90.72	0.00	9.28	0.00
0.1	0.1	2	1.99	95.07	0.00	2.61	0.00	0.00	2.31	95.08	0.00	2.63	0.00	0.00	2.29	90.50	0.00	9.50	0.00
0.1	0.3	0	0.00	95.15	2.40	0.00	1.00	0.00	2.44	95.05	2.46	0.00	1.00	0.00	2.49	97.12	2.88	0.00	1.00
0.1	0.3	0.5	0.50	94.83	2.61	2.56	0.50	0.00	0.00	94.86	2.56	2.58	0.50	0.00	0.00	95.65	1.68	2.67	0.39
0.1	0.3	1	1.00	95.18	2.46	2.34	0.51	0.00	0.00	95.20	2.47	2.33	0.51	0.00	0.00	95.86	0.54	3.60	0.13
0.1	0.3	1.5	1.50	94.66	2.65	2.69	0.50	0.00	0.00	94.70	2.65	2.65	0.50	0.00	0.00	95.50	0.00	4.50	0.00
0.1	0.3	2	2.00	94.90	0.00	2.47	0.00	0.00	2.62	94.88	0.00	2.49	0.00	0.00	2.63	95.31	0.00	4.69	0.00
0.3	0.1	0	0.00	94.70	2.88	0.00	1.00	0.98	2.01	94.79	2.83	0.00	1.00	1.01	1.99	99.43	0.57	0.00	1.00
0.3	0.1	0.5	0.49	94.79	2.45	2.53	0.49	0.00	0.00	94.69	2.45	2.55	0.49	0.00	0.00	93.73	0.00	6.27	0.00
0.3	0.1	1	0.99	94.89	2.05	2.50	0.45	0.00	0.10	94.95	2.03	2.46	0.45	0.00	0.10	91.56	0.00	8.44	0.00
0.3	0.1	1.5	1.50	95.03	1.80	2.50	0.42	0.00	0.37	95.09	1.78	2.47	0.42	0.00	0.37	91.16	0.00	8.84	0.00
0.3	0.1	2	1.98	95.17	0.00	2.28	0.00	0.00	2.55	95.17	0.00	2.25	0.00	0.00	2.58	90.80	0.00	9.20	0.00
0.3	0.3	0	0.00	94.85	2.50	0.00	1.00	0.00	2.61	94.82	2.56	0.00	1.00	0.00	2.62	97.03	2.97	0.00	1.00
0.3	0.3	0.5	0.50	95.16	2.38	2.17	0.52	0.00	0.00	95.11	2.42	2.17	0.53	0.00	0.00	96.21	1.56	2.23	0.41
0.3	0.3	1	1.00	94.98	2.47	2.55	0.49	0.00	0.00	94.99	2.45	2.56	0.49	0.00	0.00	95.66	0.66	3.68	0.15
0.3	0.3	1.5	1.50	94.76	2.68	2.56	0.51	0.00	0.00	94.78	2.70	2.52	0.52	0.00	0.00	95.48	0.00	4.52	0.00
0.3	0.3	2	2.00	94.80	0.00	2.55	0.00	0.00	2.65	94.79	0.00	2.55	0.00	0.00	2.66	95.19	0.00	4.81	0.00

^a^ DP and EP of the delta method are zero

### Application to MCTFR data

**Fig. 7:**
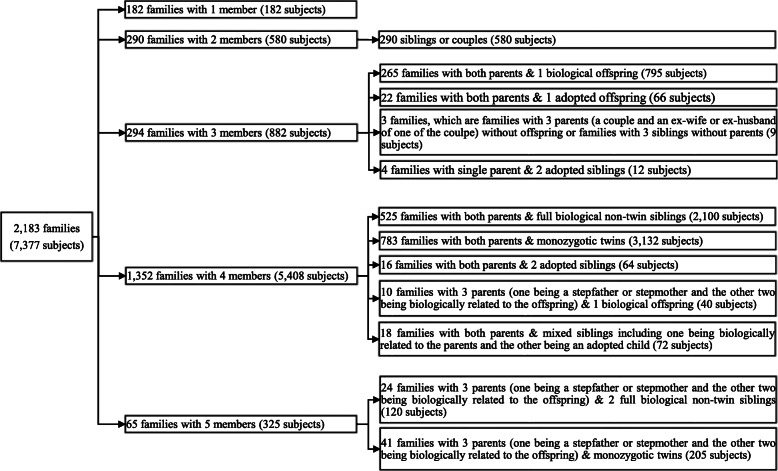
Structure of Minnesota Center for Twin and Family Research data

We applied the proposed LR, Fieller’s and delta methods to the data from the MCTFR GWAS of Behavioral Disinhibition for their practical use, and considered the following five quantitative traits: the nicotine composite score, alcohol consumption composite score, alcohol dependence composite score (DEP), illicit drug composite score (DRG) and behavioral disinhibition composite score (BD). The MCTFR data are made available for download from the database of Genotypes and Phenotypes (accession number: phs000620.v1.p1). In the MCTFR data, there are 2183 families (7377 subjects consisting of 3546 males and 3831 females), including 182 families with 1 member (182 subjects), 290 families with 2 members (580 subjects), 294 families with 3 members (882 subjects), 1352 families with 4 members (5408 subjects), and 65 families with 5 members (325 subjects). Among them, nuclear families are composed of the parents and two offspring who are monozygotic twins, full biological non-twin siblings, adopted siblings and mixed siblings with 1 biological offspring and 1 adopted offspring. Figure 7 shows more details of the family structure in the MCTFR data. Twelve thousand three hundred fifty-four single nucleotide polymorphisms (SNPs) on the X chromosome were genotyped. Note that our proposed methods are applicable in the presence of association between the SNPs and the quantitative traits of interest. So, we first conducted the association analysis for each locus and each trait. When only analyzing a single trait for all the 12,354 SNPs, the significance level was set to be *α*^′^ = 0.05/12,354 = 4.047 × 10^−6^ based on Bonferroni correction. When simultaneously analyzing multiple traits, Deng et al. [34] and McGue et al. [41] fixed the significance level at 1 × 10^−3^ for their association analysis. Therefore, in this application, we also used this significance level for the association study when simultaneously considering multiple traits. Then, we calculated the point estimate and the corresponding CIs of the skewness of XCI at the 95% confidence level for all the SNPs which are associated with a single trait at the 4.047 × 10^−6^ level or are simultaneously associated with two or more traits at the 1 × 10^−3^ level. However, we found that all these traits, and the transformed traits (e.g., log(*y* + 1)) do not satisfy the normality assumption. As such, we used the existing Levene’s test [34] to detect the association between the SNPs and the traits, which is robust to certain types of non-normal distribution.

The following quality control rules are used to filter the data. First, note that the proposed three methods for the interval estimation of *γ* only utilize unrelated females. On the other hand, although the adopted offspring in the nuclear families are biologically independent of their adopted parents, they might come from a subpopulation which is different from that of their parents. So, we deleted all the males in the data and all the offspring in the nuclear families, including the biological offspring and the adopted offspring. Second, genotyped female individuals with missing genotype rate over 10% were excluded. Third, the SNPs with missing genotype rate over 10% were deleted. Finally, we applied the PLINK software to carry out the HWE tests for SNPs [44] and the significance level is set to be 1 × 10^−4^ [45]. The SNPs with the minor allele frequency (MAF) less than 5% or those out of HWE were also excluded. As such, a total of 1955 unrelated females and 11,355 SNPs were included in this application.

The Levene’s test identified one SNP (rs17261621) which is only associated with the DRG trait at the 4.047 × 10^−6^ level, two SNPs (rs792959 and rs17261621) which are associated with both the DRG and BD traits and three SNPs (rs4825722, rs4825726 and rs2196260) which are associated with both the DEP and BD traits at the 1 × 10^−3^ level. The corresponding *P* values of the Levene’s test and the HWE test together with the position, the MAF, the point estimates and the CIs of *γ* based on the LR, Fieller’s and delta methods for these five SNPs are given in Table 5. For the DRG trait and the rs792959 locus, the point estimate of *γ*, and the 95% CIs of the LR, Fieller’s and delta methods are 2, (1.0294, 2], (1.0293, 2] and [0, 2], respectively. For the BD trait and the rs792959 locus, the point estimate of *γ*, and the corresponding 95% CIs are 2, (1.0306, 2], (1.0304, 2] and [0, 2], respectively. The CIs of the LR and Fieller’s methods for the DRG and BD traits are very similar and do not contain 1. Thus, $\hat{\gamma}$ being 2 indicates that at rs792959, 100% (2/2) of cells in heterozygous females have allele G active, and 0% of cells express allele A, which demonstrates the XCI-S towards allele G. However, the CIs of the delta method at rs792959 contain 1 (i.e., XCI-R). The conclusions drawn from the LR and Fieller’s methods here are similar to those drawn from our simulation study. However, the truncated point estimate $\hat{\gamma}$ is 2, which is the right endpoint of the interval [0, 2]. This may be because the proposed LR and Fieller’s methods require that the traits under study follow a normal distribution, while the DRG and BD traits are not normally distributed. Further, all the CIs for the other four SNPs contain 1, indicating random XCI. Particularly, for the BD trait and the rs4825722 locus, the CIs of the LR, Fieller’s and delta methods are [0, 2], which provides no information on the XCI pattern.

**Table 5:** Application of the LR, Fieller’s and delta methods to the MCTFR data for the SNPs associated with at least two traits at the 1 × 10^−3^ significance level

		Allele			*P* value				95% CI		
SNP	Position	Minor	Major	MAF	HWE test	Levene’s test	Traits	$\hat{\gamma}$	LR	Fieller	Delta
rs792959	67,891,800	G	A	0.210	0.891	7.315 × 10^−4^	DRG	2	(1.0294, 2]	(1.0293, 2]	[0, 2]
rs792959	67,891,800	G	A	0.210	0.891	7.945 × 10^−5^	BD	2	(1.0306, 2]	(1.0304, 2]	[0, 2]
rs4825722	119,728,451	A	G	0.164	0.458	6.695 × 10^−4^	DEP	1.0048	(0.2423, 2]	(0.2423, 2]	[0, 2]
rs4825722	119,728,451	A	G	0.164	0.458	8.157 × 10^−5^	BD	0.8540	[0, 2]	[0, 2]	[0, 2]
rs4825726	119,760,628	A	G	0.219	0.074	1.856 × 10^−4^	DEP	0.8474	(0.1382, 2]	(0.1381, 2]	[0, 1.8975)
rs4825726	119,760,628	A	G	0.219	0.074	1.411 × 10^−5^	BD	1.2677	(0.2400, 2]	(0.2397, 2]	[0, 2]
rs17261621	119,761,122	A	C	0.123	0.599	2.879 × 10^−6^	DRG	0.2851	[0, 1.2309)	[0, 1.2315)	[0, 0.6682)
rs17261621	119,761,122	A	C	0.123	0.599	2.310 × 10^−5^	BD	0.4834	(0.0028, 2]	(0.0025, 2]	[0, 1.1449)
rs2196260	119,761,909	G	A	0.215	0.070	5.501 × 10^−5^	DEP	0.7413	(0.0839, 2]	(0.0836, 2]	[0,1.6696)
rs2196260	119,761,909	G	A	0.215	0.070	2.486 × 10^−5^	BD	1.3667	(0.2527, 2]	(0.2523, 2]	[0, 2]

## Discussion

In this article, we extended the existing statistical measure for the degree of XCI skewing (i.e., *γ*) and the existing LR, Fieller’s and delta methods for constructing the CIs of *γ* for qualitative traits [35], and made them suitable for quantitative traits. The proposed *γ* is a ratio of two linear regression coefficients in the presence of association between the traits under study and the genotypes. According to Ma et al. [32], XCI may cause variance heterogeneity of the traits across different genotypes in females. As such, we estimated the linear regression coefficients by incorporating the information on the variance heterogeneity and then obtained the point estimate of *γ*. The Fieller’s and delta methods for calculating the CIs are simple and non-iterative procedures, while the LR method is an iterative one which needs more computing time. On the other hand, the hypothesis testing of the LR, Fieller’s and delta methods was also investigated. We conducted extensive simulation studies (two different values of additive effect, two groups of allele frequencies, five different values of *γ*, two different sample sizes, and two different values of inbreeding coefficient) to assess the validity of the proposed methods. Simulation results demonstrate that the median of the point estimates of *γ* is very close to the pre-specified true value of *γ*. The LR and Fieller’s methods have similar performance in the CP, ML/(ML + MR), DP and EP. The CPs of both methods are controlled around 95% for all the simulated scenarios, and the values of the ML/(ML + MR) for both methods generally maintain close to 0.5, except for the cases of *p* = 0.1 and *n* = 1,000, and the situations of *γ* = 0 and 2. Besides, both methods perform better than the delta method in the CP and ML/(ML + MR). On the other hand, the LR and Fieller’s methods control the size well and almost have the same test powers. However, the type I error rate of the delta method is inflated or conservative under most simulation settings. This may be because the distribution of the point estimate $\hat{\gamma}$ is asymmetric after being cut off by 0 and 2, and then $\frac{\hat{\gamma}-{\gamma}_0}{\sqrt{\hat{\mathrm{Var}}\left(\hat{\gamma}\right)}}\sim N\left(0,1\right)$ is not so strictly correct. Another possible reason why the delta method performs so poorly is that the first order Taylor expansion of $\hat{\gamma}$ does not suffice. To investigate the performance of the delta method with higher order Taylor expansion, we used the second order Taylor expansion of $\hat{\gamma}$ to calculate the asymptotic variance of $\hat{\gamma}$, which can be implemented in the “propagate” package in R software [46]. However, most of the estimated type I error rates for the delta method are still inflated or conservative, even though they appear to be controlled better than those in Tables 1 and 2 (data not shown for brevity). Therefore, in practical use, we recommend the Fieller’s method because it is a non-iterative procedure and has the similar performance to the LR method.

So far, we are not aware of any association study for the X-chromosomal SNPs in the MCTFR data. In fact, we also found that all the five traits in the MCTFR data are not normally distributed. On the other hand, when simultaneously analyzing multiple traits for the X-chromosomal SNPs, Deng et al. [34] fixed the significance level at 1 × 10^−3^ for their association analysis. So, in our real data application, we used the existing Levene’s test [34] to test for the association between the X-chromosomal SNPs and the five traits at the significance level of 1 × 10^−3^, which does not require the normality assumption for the traits. However, when only analyzing a single trait for all the 12,354 SNPs, the significance level is set to be *α*^′^ = 0.05/12,354 = 4.047 × 10^−6^ based on Bonferroni correction. One SNP (rs17261621) is shown to be only associated with the DRG trait at the 4.047 × 10^−6^ level, two SNPs (rs792959 and rs17261621) are identified to be associated with both the DRG and BD traits, and three SNPs (rs4825722, rs4825726 and rs2196260) are found to be associated with both the DEP and BD traits at the 1 × 10^−3^ level. In addition, we applied the proposed LR, Fieller’s and delta methods to these five SNPs and calculated the CIs of the skewness of XCI at the 95% confidence level. The CIs based on the LR and Fieller’s methods show that only rs792959 undergoes XCI-S. However, these conclusions need to be further confirmed by molecular genetics. On the other hand, the proposed LR and Fieller’s methods require that the traits under study follow a normal distribution, while the DEP, DRG and BD traits are not normally distributed. Since we have no suitable data of this kind available, it is of future interest to apply the three proposed methods to datasets with traits following normal distributions and to further confirm their practical use.

Besides, the proposed methods have the following issues to discuss. First, to make the point estimate and the CIs of *γ* more interpretable, we simply use the interval [0, 2] to truncate the original point estimate and the original CIs, which may cause potential loss of information, and may also lead to the truncated CIs being empty sets when the original CIs lie outside [0, 2]. Fortunately, from our simulation study, the proportion of the CIs being empty sets or being reduced to be a point among all the simulation replications is all less than 2.7%. On the other hand, to incorporate the interval constraint of [0, 2] into statistical inference, we will develop a future Bayesian method to estimate the skewness of XCI by considering such constraint as prior information. Second, the proposed methods require the association between the traits and the SNPs being present. As such, in genome-wide association study, we could regard the screening of the associated SNPs as a preliminary step before estimating the skewness of XCI. If such association is not statistically significant, the LR and Fieller’s methods may result in the discontinuous CIs, which is difficult to interpret. Third, the normality assumption of the traits under study is needed in the proposed methods. In future, we will extend them to accommodate the traits not normally distributed. Finally, the proposed methods are only applicable to unrelated female subjects. Thus, we will extend the proposed methods and make them suitable for data with family or pedigree structure in future studies.

## Conclusions

We recommend the Fieller’s method in practical use because it is a non-iterative procedure and almost has the same performance as the LR method. On the other hand, only rs792959, which is identified to be associated with both the DRG and BD traits, may undergo XCI-S, which needs to be confirmed by molecular genetics.

## Methods

### Notations and point estimate of ***γ***

Consider an X-linked diallelic QTL. Let *D* and *d* represent the mutant allele and the normal allele at the QTL with the frequencies being *p* and *q* (*p* + *q* = 1), respectively. Note that XCI is unrelated to males and we only consider females here. Then, there are three possible genotypes *dd*, *Dd* and *DD* at the QTL in females. The corresponding frequencies are *g*_0_ = *q*^2^ + *ρpq*, *g*_1_ = 2(1 − *ρ*)*pq* and *g*_2_ = *p*^2^ + *ρpq*, respectively, where *ρ* is the inbreeding coefficient. *ρ* = 0 means that Hardy-Weinberg equilibrium (HWE) holds in the female population, while *ρ* ≠ 0 denotes Hardy-Weinberg disequilibrium. Suppose that *Y* and *G* are the value of the quantitative trait under study and the genotype of a female subject, respectively. Notice that XCI may lead to variance heterogeneity of *Y* across different genotypes [32]. So, we assume that ${\left.Y\right|}_{G= dd}\sim N\left({\mu}_0,{\sigma}_0^2\right)$, ${\left.Y\right|}_{G= Dd}\sim N\left({\mu}_1,{\sigma}_1^2\right)$ and ${\left.Y\right|}_{G= DD}\sim N\left({\mu}_2,{\sigma}_2^2\right).$ Further, let *X*_1_ = *I*_{*G* = *Dd* or *DD*}_ and *X*_2_ = *I*_{*G* = *DD*}_. As such, *X*_1_ denotes that this female carries at least one mutant allele *D* and *X*_2_ indicates that she is a homozygote *DD*. Then, to construct the statistical measure of the skewness of XCI, we consider the following linear regression model

1 $$ E\left(Y\right|{X}_1,{X}_2,\boldsymbol{Z}\Big)={\beta}_0+{\beta}_1{X}_1+{\beta}_2{X}_2+{\boldsymbol{b}}^{\mathrm{T}}\boldsymbol{Z}, $$

where ***Z*** is a vector of covariates which need to be adjusted, *β*_0_ is the intercept, *β*_1_ and *β*_2_ respectively are the regression coefficients of *X*_1_ and *X*_2_, and ***b*** is a vector of the regression coefficients for ***Z***. From Eq. (1), we have *μ*_0_ = *β*_0_ + ***b***^T^***Z***, *μ*_1_ = *β*_0_ + *β*_1_ + ***b***^T^***Z*** and *μ*_2_ = *β*_0_ + *β*_1_ + *β*_2_ + ***b***^T^***Z***. Under XCI-R, *μ*_1_ should lie midway between *μ*_0_ and *μ*_2_, which is ${\beta}_0+\frac{\beta_1+{\beta}_2}{2}+{\boldsymbol{b}}^{\mathrm{T}}\boldsymbol{Z}$. Hence, for heterozygous females, any statistically significant deviation from such value can be regarded as an evidence of XCI-S. This is equivalent to that $\frac{\mu_1-{\mu}_0}{\mu_2-{\mu}_0}=\frac{\beta_1}{\beta_1+{\beta}_2}$ is far away from 0.5. Therefore, we define the following parameter *γ* to measure the skewness of XCI

2 $$ \gamma =\frac{2{\beta}_1}{\beta_1+{\beta}_2},\gamma \in \left[0,2\right], $$

with *β*_1_ + *β*_2_ ≠ 0. And *θ* = *γ*/2, on the average, is indicative of the proportion of cells in a *Dd* female keeping the mutant allele *D* active. Thus, *γ* = 1 denotes XCI-R. 1 < *γ* ≤ 2 means the XCI-S towards *D* and 0 ≤ *γ* < 1 represents the XCI-S against *D*. For example, when *γ* = 1.5, then *θ* = 75%, which means that 75% cells have mutant allele *D* active and the other 25% cells express the normal allele *d*.

Let *β* = (*β*_1_ + *β*_2_)/2, then *γ* = *β*_1_/*β*. In this regard, we obtain *β*_1_ = *γβ* and *β*_2_ = (2 − *γ*)*β*, where *β* ≠ 0. Let *X* = *γX*_1_ + (2 − *γ*)*X*_2_, then Eq. (1) becomes

3 $$ E\left(Y\right|{X}_1,{X}_2,\boldsymbol{Z}\Big)={\beta}_0+\gamma \beta {X}_1+\left(2-\gamma \right)\beta {X}_2+{\boldsymbol{b}}^{\mathrm{T}}\boldsymbol{Z}={\beta}_0+\beta X+{\boldsymbol{b}}^{\mathrm{T}}\boldsymbol{Z}. $$

Here, the genotypic value *X* equals 0, *γ* and 2 for genotypes *dd*, *Dd* and *DD*, respectively, which implies that the definition of *γ* coincides with the coding strategy of Wang et al. [27] for XCI. On the other hand, from Eq. (2), we observe that *γ* can be well defined when the association between *Y* and the allele of interest is present (i.e., *β* ≠ 0).

Assume that we collect a sample of *n* independent females. Let *n*_0_, *n*_1_ and *n*_2_ be the number of females with genotypes *dd*, *Dd* and *DD*, respectively. So, *n*_0_ + *n*_1_ + *n*_2_ = *n*. Let *y*_*ij*_, *x*_*ij*1_, *x*_*ij*2_ and ***z***_*ij*_ denote the values of *Y*, *X*_1_, *X*_2_ and ***Z*** of female *j* (*j* = 1, 2, ⋯, *n*_*i*_), where *i* = 0, 1, 2 respectively correspond to genotypes *dd*, *Dd* and *DD*. According to Eq. (1), the log-likelihood function of the sample is

$$ {\displaystyle \begin{array}{c}{l}_1\left({\beta}_0,{\beta}_1,{\beta}_2,{\sigma}_0,{\sigma}_1,{\sigma}_2,\boldsymbol{b}\right)=-{\sum}_{i=0}^2{n}_i\log {\sigma}_i-{\sum}_{j=1}^{n_0}\frac{{\left({y}_{0j}-{\beta}_0-{\boldsymbol{b}}^{\mathrm{T}}{\boldsymbol{z}}_{0j}\right)}^2}{2{\sigma}_0^2}\\ {}-{\sum}_{j=1}^{n_1}\frac{{\left({y}_{1j}-{\beta}_0-{\beta}_1-{\boldsymbol{b}}^{\mathrm{T}}{\boldsymbol{z}}_{1j}\right)}^2}{2{\sigma}_1^2}\\ {}-{\sum}_{j=1}^{n_2}\frac{{\left({y}_{2j}-{\beta}_0-{\beta}_1-{\beta}_2-{\boldsymbol{b}}^{\mathrm{T}}{\boldsymbol{z}}_{2j}\right)}^2}{2{\sigma}_2^2}-n\log \left(\sqrt{2\pi}\right).\end{array}} $$

Then, by maximizing the above equation, the maximum likelihood estimates ${\hat{\beta}}_0,{\hat{\beta}}_1,{\hat{\beta}}_2,{\hat{\sigma}}_0,{\hat{\sigma}}_1,{\hat{\sigma}}_2$ and $\hat{\boldsymbol{b}}$ of *β*_0_, *β*_1_, *β*_2_, *σ*_0_, *σ*_1_, *σ*_2_ and ***b*** can be derived. As such, according to Eq. (2), the point estimate of *γ* can be given as $\frac{2{\hat{\beta}}_1}{{\hat{\beta}}_1+{\hat{\beta}}_2}$. Notice that *γ* is bounded in [0, 2]. Then, the final point estimate of *γ* is cut off by 0 and 2, and denoted by $\hat{\gamma}$.

### Confidence interval of ***γ***

Here, we extend the three methods proposed by Wang et al. [35] to construct the CI of *γ* to quantitative traits as follows. To obtain the CI of *γ* based on the LR method, we first develop a likelihood ratio test for testing the null hypothesis *H*_0_ : *γ* = *γ*_0_ below, where *γ*_0_ ∈ [0, 2] is a pre-specified constant, e.g., *γ*_0_ = 1 (XCI-R). From Eq. (3), the log-likelihood function of the sample under *H*_0_ is

$$ {\displaystyle \begin{array}{c}{l}_0\left({\beta}_0,\beta, {\sigma}_0,{\sigma}_1,{\sigma}_2,\boldsymbol{b}\right)=-{\sum}_{i=0}^2{n}_i\log {\sigma}_i-{\sum}_{j=1}^{n_0}\frac{{\left({y}_{0j}-{\beta}_0-{\boldsymbol{b}}^{\mathrm{T}}{\boldsymbol{z}}_{0j}\right)}^2}{2{\sigma}_0^2}\\ {}-{\sum}_{j=1}^{n_1}\frac{{\left({y}_{1j}-{\beta}_0-{\gamma}_0\beta -{\boldsymbol{b}}^{\mathrm{T}}{\boldsymbol{z}}_{1j}\right)}^2}{2{\sigma}_1^2}\\ {}-{\sum}_{j=1}^{n_2}\frac{{\left({y}_{2j}-{\beta}_0-2\beta -{\boldsymbol{b}}^{\mathrm{T}}{\boldsymbol{z}}_{2j}\right)}^2}{2{\sigma}_2^2}-n\log \left(\sqrt{2\pi}\right).\end{array}} $$

By maximizing the above equation, the maximum likelihood estimates ${\overset{\sim }{\beta}}_0,\overset{\sim }{\beta },{\overset{\sim }{\sigma}}_0,{\overset{\sim }{\sigma}}_1,{\overset{\sim }{\sigma}}_2$ and $\overset{\sim }{\boldsymbol{b}}$ of *β*_0_, *β*, *σ*_0_, *σ*_1_, *σ*_2_ and ***b*** under *H*_0_ can be obtained. So, the likelihood ratio test is

$$ \lambda \left({\gamma}_0\right)=2\left({l}_1\left({\hat{\beta}}_0,{\hat{\beta}}_1,{\hat{\beta}}_2,{\hat{\sigma}}_0,{\hat{\sigma}}_1,{\hat{\sigma}}_2,\hat{\boldsymbol{b}}\right)-{l}_0\left({\overset{\sim }{\beta}}_0,\overset{\sim }{\beta },{\overset{\sim }{\sigma}}_0,{\overset{\sim }{\sigma}}_1,{\overset{\sim }{\sigma}}_2,\overset{\sim }{\boldsymbol{b}}\right)\right), $$

which asymptotically follows the chi-square distribution with the degree of freedom being one (i.e., ${\chi}_1^2$).

Then, the 100(1 − *α*)% CI of *γ* based on *λ*(*γ*_0_) is $\left\{{\gamma}_0:P\left(\lambda \left({\gamma}_0\right)<{\chi}_{1-\alpha, 1}^2\right)\right\}$ and the confidence limits satisfy

4 $$ f\left({\gamma}_0\right)=\lambda \left({\gamma}_0\right)-{\chi}_{1-\alpha, 1}^2=0. $$

That is, the 100(1 − *α*)% CI of *γ* is the interval satisfying *f*(*γ*_0_) < 0. Note that *γ* should be bounded in [0, 2]. As such, the bisection method is applied to find all the roots of Eq. (4) within [0, 2] by using the “rootSolve” package in R software [46]. This indicates that the LR method is an iterative procedure. If Eq. (4) has no root in [0, 2] and *f*(*γ*_0_) < 0, the CI is taken to be [0, 2]. On the contrary, when Eq. (4) has no root in [0, 2], but *f*(*γ*_0_) > 0, the resulting CI is an empty set. When Eq. (4) has only one root *γ*^*LR*^ in [0, 2] and *f*(*γ*_0_) ≥ 0, the CI is reduced to be a point. When Eq. (4) has only one root *γ*^*LR*^ in [0, 2] and *f*(0)*f*(2) < 0, there are two different situations. If *f*(0) > 0 and *f*(2) < 0, then *f*(*γ*_0_) < 0 will be satisfied within (*γ*^*LR*^, 2] and the CI is taken as (*γ*^*LR*^, 2]; otherwise, the CI is set to be [0, *γ*^*LR*^). When Eq. (4) has two unequal roots ${\gamma}_L^{LR}$ and ${\gamma}_U^{LR}$ in [0, 2] with ${\gamma}_L^{LR}<{\gamma}_U^{LR}$, *f*(*γ*_0_) < 0, ${\gamma}_0\in \left({\gamma}_L^{LR},{\gamma}_U^{LR}\right)$ means that the CI is $\left({\gamma}_L^{LR},{\gamma}_U^{LR}\right)$. Otherwise, the CI is $\left[0,{\gamma}_L^{LR}\right)\cup \left({\gamma}_U^{LR},2\right]$, the union of two disjoint intervals, which is a discontinuous CI.

Since $\hat{\gamma}$ is a ratio estimate, borrowing the idea of Wang et al. [35], we find that the standard error of $\hat{\gamma}$ can be approximated by using the delta method. Specifically, take a first order Taylor expansion of *γ* around the point (*β*_1_, *β*) and evaluate it at $\left({\hat{\beta}}_1,\hat{\beta}\right)$, which yields $\hat{\gamma}\approx \frac{\beta_1}{\beta }+\left({\hat{\beta}}_1-{\beta}_1\right)\frac{1}{\beta }-\left(\hat{\beta}-\beta \right)\frac{\beta_1}{\beta^2}$, where $\hat{\beta}=\frac{{\hat{\beta}}_1+{\hat{\beta}}_2}{2}$. Then,

5 $$ \mathrm{Var}\left(\hat{\gamma}\right)=\frac{1}{\beta^2}\mathrm{Var}\left({\hat{\beta}}_1\right)+\frac{\beta_1^2}{\beta^4}\mathrm{Var}\left(\hat{\beta}\right)-\frac{2{\beta}_1}{\beta^3}\mathrm{Cov}\left({\hat{\beta}}_1,\hat{\beta}\right), $$

where $\mathrm{Var}\left(\hat{\beta}\right)=\mathrm{Var}\left(\frac{{\hat{\beta}}_1+{\hat{\beta}}_2}{2}\right)=\frac{1}{4}\left[\mathrm{Var}\left({\hat{\beta}}_1\right)+\mathrm{Var}\left({\hat{\beta}}_2\right)+2\mathrm{Cov}\left({\hat{\beta}}_1,{\hat{\beta}}_2\right)\right]$ and $\mathrm{Cov}\left({\hat{\beta}}_1,\hat{\beta}\right)=\mathrm{Cov}\left({\hat{\beta}}_1,\frac{{\hat{\beta}}_1+{\hat{\beta}}_2}{2}\right)=\frac{1}{2}\mathrm{Var}\left({\hat{\beta}}_1\right)+\frac{1}{2}\mathrm{Cov}\left({\hat{\beta}}_1,{\hat{\beta}}_2\right)$. Here, $\mathrm{Var}\left({\hat{\beta}}_1\right)$, $\mathrm{Var}\left({\hat{\beta}}_2\right)$ and $\mathrm{Cov}\left({\hat{\beta}}_1,{\hat{\beta}}_2\right)$ are the elements of the variance-covariance matrix of ${\hat{\beta}}_1$ and ${\hat{\beta}}_2$, which can be computed by using the “glm” function in R software [46]. Respctively replacing *β*_1_ and *β* by ${\hat{\beta}}_1$ and $\hat{\beta}$ in Eq. (5), we get the estimate of the variance of $\hat{\gamma}$ as $\hat{\mathrm{Var}}\left(\hat{\gamma}\right)=\frac{1}{{\hat{\beta}}^2}\mathrm{Var}\left({\hat{\beta}}_1\right)+\frac{{\hat{\beta}}_1^2}{{\hat{\beta}}^4}\mathrm{Var}\left(\hat{\beta}\right)-\frac{2{\hat{\beta}}_1}{{\hat{\beta}}^3}\mathrm{Cov}\left({\hat{\beta}}_1,\hat{\beta}\right)$. From the fact that $\frac{\hat{\gamma}-{\gamma}_0}{\sqrt{\hat{\mathrm{Var}}\left(\hat{\gamma}\right)}}\sim N\left(0,1\right)$ asymptotically, the 100(1 − *α*)% CI of *γ* based on the delta method is $\left(\hat{\gamma}-{z}_{\alpha /2}\sqrt{\hat{\mathrm{Var}}\Big(\hat{\gamma}}\Big),\hat{\gamma}+{z}_{\alpha /2}\sqrt{\hat{\mathrm{Var}}\Big(\hat{\gamma}}\Big)\right)\cap \left[0,2\right]$, where *z*_*α*/2_ is the upper *α*/2 quantile of the standard normal distribution.

Now, we consider the CI of *γ* based on the Fieller’s method, just like Wang et al. [35]. Under *H*_0_ : *γ* = *γ*_0_, we have *β*_1_ − *γ*_0_*β* = 0. Then, we can construct the following Wald test for testing *H*_0_ : *γ* = *γ*_0_

$$ \frac{{\hat{\beta}}_1-{\gamma}_0\hat{\beta}}{\sqrt{\mathrm{Var}\left({\hat{\beta}}_1\right)+{\gamma}_0^2\mathrm{Var}\left(\hat{\beta}\right)-2{\gamma}_0\mathrm{Cov}\left({\hat{\beta}}_1,\hat{\beta}\right)}}\sim N\left(0,1\right). $$

The confidence limits of the 100(1 − *α*)% CI based on the Fieller’s method satisfy $\frac{{\hat{\beta}}_1-{\gamma}_0\hat{\beta}}{\sqrt{\mathrm{Var}\left({\hat{\beta}}_1\right)+{\gamma}_0^2\mathrm{Var}\left(\hat{\beta}\right)-2{\gamma}_0\mathrm{Cov}\left({\hat{\beta}}_1,\hat{\beta}\right)}}={z}_{\alpha /2}$, which is equivalent to the quadratic equation $A{\gamma}_0^2+B{\gamma}_0+C=0$ with respect to *γ*_0_. Here, $A={\hat{\beta}}^2-{z}_{\alpha /2}^2\mathrm{Var}\left(\hat{\beta}\right)$, $B=2{z}_{\alpha /2}^2\mathrm{Cov}\left({\hat{\beta}}_1,\hat{\beta}\right)-2{\hat{\beta}}_1\hat{\beta}$ and $C={{\hat{\beta}}_1}^2-{z}_{\alpha /2}^2\mathrm{Var}\left({\hat{\beta}}_1\right)$. Assume that *Δ* = *B*^2^ − 4*AC*. From Fieller’s theorem, *A* > 0 implies *Δ* > 0. Further, *A* > 0 and $\left|\hat{\beta}/\sqrt{\mathrm{Var}\left(\hat{\beta}\right)}\right|>{z}_{\alpha /2}$ are equivalent to each other, which mean that the association is present at the significance level of *α*. In addition, when *Δ* = 0 or *A* = 0, the CI is reduced to a point. As such, the 100(1 − *α*)% CI based on the Fieller’s method is

$$ \left\{\begin{array}{cc}\left(\frac{-B-\sqrt{\Delta}}{2A},\frac{-B+\sqrt{\Delta}}{2A}\right)\cap \left[0,2\right],& \mathrm{if}\ \varDelta >0\ \mathrm{and}\ A>0\ \\ {}\left(\left(-\infty, \frac{-B+\sqrt{\Delta}}{2A}\right)\cup \left(\frac{-B-\sqrt{\Delta}}{2A},+\infty \right)\right)\cap \left[0,2\right],& \mathrm{if}\ \varDelta >0\ \mathrm{and}\ A<0\ \\ {}\left[0,2\right],& \mathrm{if}\ \varDelta <0\ \mathrm{and}\ A<0\ \end{array}\right.. $$

It should be noted that if *Δ* > 0, the above CI may be an empty set. When *Δ* > 0 and *A* < 0, the corresponding CI may be the union of two disjoint intervals, which is the discontinuous CI.

### Simulation settings

For simplicity, we do not include any covariate in the model. The frequency *p* of allele *D* at the locus on X chromosome is fixed at 0.1 and 0.3. The inbreeding coefficient *ρ* is taken as 0 and 0.05 to respectively simulate the situation of HWE and that of Hardy-Weinberg disequilibrium. Let *β*_0_ = 0.1 and *β* = 0.3. Further, *β*_1_ = *γβ* and *β*_2_ = (2 − *γ*)*β* are calculated from *β* = 0.3 and *γ*, where *γ* takes values of 0, 0.5, 1, 1.5 and 2. *γ*_0_ is also assigned to be 0, 0.5, 1, 1.5 and 2 with *γ* = *γ*_0_ to simulate the type I error rates of the proposed methods and *γ* ≠ *γ*_0_ for simulating the their test powers. As mentioned in Ma et al. [32], the variance ${\sigma}_1^2$ of the trait value for heterozygous females is generally larger than ${\sigma}_0^2$ and ${\sigma}_2^2$ for homozygous females due to XCI. So, we set ${\sigma}_0^2={\sigma}_2^2=1$, and ${\sigma}_1^2=\theta \left(1-\theta \right){a}^2+1.1$, where *a* is the additive effect of the QTL, *θ* = *γ*/2 is the inactivation ratio as mentioned before, and the variance caused by other factors is fixed to be 1.1. Here, *a* is set to be 0.1 and 0.3. The sample size *n* is selected to be 1,000 and 2,000. The genotype of each female is simulated according to the allele frequency *p* and the inbreeding coefficient *ρ*. Then, the trait value *Y* of this female given her genotype is generated by ${\left.Y\right|}_{G= dd}\sim N\left({\beta}_0,{\sigma}_0^2\right)$, ${\left.Y\right|}_{G= Dd}\sim N\left({\beta}_0+{\beta}_1,{\sigma}_1^2\right)$ or ${\left.Y\right|}_{G= DD}\sim N\left({\beta}_0+{\beta}_1+{\beta}_2,{\sigma}_2^2\right)$. For each simulation setting, the simulations are conducted based on *K* = 10,000 replications and the significance level *α* is fixed at 5%. The simulation study is implemented in R software (version 3.2.5) [46].

Notice that the distribution of the point estimate $\hat{\gamma}$ may be asymmetric. So, we list the median of $\hat{\gamma}$ ’s over *K* replications to describe the central tendency of this skewed distribution. We assess the statistical properties of the CIs of *γ* by the following indexes. The coverage probability (CP) is the proportion that the CIs contain the true value *γ* among *K* replications, irrespective of the CI being continuous or discontinuous. DP and EP are the proportion of the discontinuous CIs and that of the CIs being an empty set or being reduced to be a point among *K* replications, respectively. Simulation study is also carried out to investigate the probabilities of the CI missing the true value *γ* on the left (ML) and on the right (MR), and the value of the ratio ML/(ML + MR), which is close to 0.5 when the balance between ML and MR is achieved. Here, $\mathrm{ML}=\frac{\#\left[\left(\gamma <{\gamma}_L\right)\cap \left({\gamma}_L\le \hat{\gamma}\le {\gamma}_U\right)\right]}{K}$ and $\mathrm{MR}=\frac{\#\left[\left(\gamma >{\gamma}_U\right)\cap \left({\gamma}_L\le \hat{\gamma}\le {\gamma}_U\right)\right]}{K}$, where # is the counting measure and (*γ*_*L*_, *γ*_*U*_) ’s are the continuous CIs. We only consider the continuous CIs when computing the ML and MR, because we cannot distinguish between the left side and the right side of the discontinuous CIs.

## Supplementary Information

**Additional file 1: Tables S1-S2.** Estimated sizes for testing *H*_0_ : *γ* = *γ*_0_ for the LR, Fieller’s and delta methods with *a* = 0.1 and 0.3, *p* = 0.1 and 0.3, and *ρ* = 0.05 based on 10,000 replicates and 5% significance level when *n* = 1,000 and 2,000, respectively. **Tables S3-S4.** Estimated median of the point estimates of *γ*, CP, ML, MR, ML/(ML + MR), DP and EP of two-sided 95% CIs of *γ* for the LR, Fieller’s and delta methods against *γ*, with *a* = 0.1 and 0.3, *p* = 0.1 and 0.3, and *ρ* = 0.05 based on 10,000 replicates when *n* = 1,000 and 2,000, respectively. **Figures S1-S2.** Estimated powers for the LR and Fieller’s methods against *γ* based on 10,000 replicates and 5% significance level with *a* = 0.1 and 0.3, *p* = 0.1 and 0.3, *ρ* = 0 and *n* = 1,000 when *γ*_0_ = 0.5 and 1.5, respectively. **Figures S3-S7.** Estimated powers for the LR and Fieller’s methods against *γ* based on 10,000 replicates and 5% significance level with *a* = 0.1 and 0.3, *p* = 0.1 and 0.3, *ρ* = 0.05 and *n* = 1,000 when *γ*_0_ = 0, 0.5, 1, 1.5 and 2, respectively. **Figures S8-S9.** Estimated powers for the LR and Fieller’s methods against *γ* based on 10,000 replicates and 5% significance level with *a* = 0.1 and 0.3, *p* = 0.1 and 0.3, *ρ* = 0 and *n* = 2,000 when *γ*_0_ = 0.5 and 1.5, respectively. **Figures S10-S14.** Estimated powers for the LR and Fieller’s methods against *γ* based on 10,000 replicates and 5% significance level with *a* = 0.1 and 0.3, *p* = 0.1 and 0.3, *ρ* = 0.05 and *n* = 2,000 when *γ*_0_ = 0, 0.5, 1, 1.5 and 2, respectively.

## Data Availability

The Minnesota Center for Twin and Family Research data used for the analyses described in this article can be found on the database of Genotypes and Phenotypes with accession number phs000620.v1.p1 (https://www.ncbi.nlm.nih.gov/projects/gap/cgi-bin/study.cgi?study_id=phs000620.v1.p1).

## Abbreviations

BD: Behavioral disinhibition composite score
CI: Confidence interval
CP: Coverage probability
DEP: Alcohol dependence composite score
DP: Proportion of the discontinuous CIs
DRG: Illicit drug composite score
EP: Proportion of the CIs being empty sets or being reduced to be a point
GWAS: Genome-wide association study
HWE: Hardy-Weinberg equilibrium
LR: Likelihood ratio
MAF: Minor allele frequency
MCTFR: Minnesota Center for Twin and Family Research
ML: Left tail error
MR: Right tail error
QTL: Quantitative trait loci
SNP: Single nucleotide polymorphism
XCI: X chromosome inactivation
XCI-E: Escape from X chromosome inactivation
XCI-R: Random X chromosome inactivation
XCI-S: Skewed X chromosome inactivation
