# Supplementary material for: A statistical measure for the skewness of X chromosome inactivation for quantitative traits and its application to the MCTFR data
Source: BMC Genom Data. 2021 Jul 2;22:24. doi: 10.1186/s12863-021-00978-z (PMC8254321; doi:10.1186/s12863-021-00978-z)
Supplement: Supplementary file 1 — Additional file 1: Tables S1-S2. Estimated sizes for testing H0 : γ = γ0 for the LR, Fieller’s and delta methods with a = 0.1 and 0.3, p = 0.1 and 0.3, and ρ = 0.05 based on 10,000 replicates and 5% significance level when n = 1,000 and 2,000, respectively. Tables S3-S4. Estimated median of the point estimates of γ, CP, ML, MR, ML/(ML + MR), DP and EP of two-sided 95% CIs of γ for the LR, Fieller’s and delta methods against γ, with a = 0.1 and 0.3, p = 0.1 and 0.3, and ρ = 0.05 based on 10,000 replicates when n = 1,000 and 2,000, respectively. Figures S1-S2. Estimated powers for the LR and Fieller’s methods against γ based on 10,000 replicates and 5% significance level with a = 0.1 and 0.3, p = 0.1 and 0.3, ρ = 0 and n = 1,000 when γ0 = 0.5 and 1.5, respectively. Figures S3-S7. Estimated powers for the LR and Fieller’s methods against γ based on 10,000 replicates and 5% significance level with a = 0.1 and 0.3, p = 0.1 and 0.3, ρ = 0.05 and n = 1,000 when γ0 = 0, 0.5, 1, 1.5 and 2, respectively. Figures S8-S9. Estimated powers for the LR and Fieller’s methods against γ based on 10,000 replicates and 5% significance level with a = 0.1 and 0.3, p = 0.1 and 0.3, ρ = 0 and n = 2,000 when γ0 = 0.5 and 1.5, respectively. Figures S10-S14. Estimated powers for the LR and Fieller’s methods against γ based on 10,000 replicates and 5% significance level with a = 0.1 and 0.3, p = 0.1 and 0.3, ρ = 0.05 and n = 2,000 when γ0 = 0, 0.5, 1, 1.5 and 2, respectively. [file 12863_2021_978_MOESM1_ESM.pdf]

Additional file

Table S1 Estimated sizes (in %) for testing H_0: $\gamma = \gamma_0$ for the LR, Fieller's and delta methods with $a = 0.1$ and 0.3, $p = 0.1$ and 0.3, $n = 1{,}000$ and $\rho = 0.05$ based on $10{,}000$ replicates and 5% significance level

a	p	γ_0	LR	Fieller	Delta
0.1	0.1	0	4.99	4.98	0.30
0.1	0.1	0.5	4.94	4.81	5.89
0.1	0.1	1	5.26	5.13	9.85
0.1	0.1	1.5	4.83	4.76	10.47
0.1	0.1	2	4.75	4.77	11.30
0.1	0.3	0	4.90	5.01	2.94
0.1	0.3	0.5	5.16	5.04	3.08
0.1	0.3	1	5.14	5.22	3.98
0.1	0.3	1.5	5.18	5.10	5.30
0.1	0.3	2	4.75	4.78	5.65
0.3	0.1	0	5.03	4.98	0.30
0.3	0.1	0.5	4.91	4.93	6.07
0.3	0.1	1	5.19	5.11	10.69
0.3	0.1	1.5	4.81	4.77	10.78
0.3	0.1	2	4.86	4.96	10.75
0.3	0.3	0	5.10	5.15	2.89
0.3	0.3	0.5	5.12	5.04	3.07
0.3	0.3	1	4.70	4.69	3.97
0.3	0.3	1.5	5.05	5.04	4.90
0.3	0.3	2	4.70	4.80	5.45

Table S2 Estimated sizes (in %) for testing H_0: $\gamma = \gamma_0$ for the LR, Fieller's and delta methods with $a = 0.1$ and 0.3, $p = 0.1$ and 0.3, $n = 2{,}000$ and $\rho = 0.05$ based on $10{,}000$ replicates and 5% significance level

a	p	γ_0	LR	Fieller	Delta
0.1	0.1	0	5.11	5.15	0.57
0.1	0.1	0.5	5.00	4.88	5.92
0.1	0.1	1	4.97	4.94	7.97
0.1	0.1	1.5	4.96	4.91	8.88
0.1	0.1	2	4.91	4.80	8.17
0.1	0.3	0	4.82	4.84	2.65
0.1	0.3	0.5	5.15	5.19	4.19
0.1	0.3	1	5.07	5.04	4.34
0.1	0.3	1.5	5.06	5.03	4.53
0.1	0.3	2	5.14	5.07	4.80
0.3	0.1	0	4.85	4.73	0.53
0.3	0.1	0.5	4.86	4.84	6.04
0.3	0.1	1	5.10	5.11	8.60
0.3	0.1	1.5	5.12	5.16	8.71
0.3	0.1	2	4.75	4.80	8.97
0.3	0.3	0	5.07	4.98	2.86
0.3	0.3	0.5	4.85	4.94	4.26
0.3	0.3	1	5.04	5.04	4.53
0.3	0.3	1.5	5.02	4.96	4.45
0.3	0.3	2	5.11	5.09	4.57

Table S3 Estimated median of the point estimates of γ, CP (in %), ML (in %), MR (in %), Ratio $(\mathrm{ML}/(\mathrm{ML}+\mathrm{MR}))$, DP (in %) and EP (in %) of two-sided 95% CIs of γ for the LR, Fieller's and delta methods against γ, with $a = 0.1$ and 0.3, $p = 0.1$ and 0.3, $n = 1{,}000$ and $\rho = 0.05$ based on $10{,}000$ replicates

				LR						Fieller						Delta[a]			
a	p	γ	Median	CP	ML	MR	Ratio	DP	EP	CP	ML	MR	Ratio	DP	EP	CP	ML	MR	Ratio
0.1	0.1	0	0.00	95.01	2.62	0.00	1.00	1.83	1.51	95.02	2.61	0.00	1.00	1.88	1.50	99.70	0.30	0.00	1.00
0.1	0.1	0.5	0.47	95.06	1.59	2.56	0.38	0.13	0.05	95.19	1.55	2.45	0.39	0.18	0.05	94.11	0.00	5.89	0.00
0.1	0.1	1	0.96	94.74	1.09	2.56	0.30	0.05	0.19	94.87	1.08	2.49	0.30	0.04	0.18	90.15	0.00	9.85	0.00
0.1	0.1	1.5	1.44	95.17	0.79	2.37	0.25	0.00	0.69	95.24	0.77	2.35	0.25	0.00	0.73	89.53	0.00	10.47	0.00
0.1	0.1	2	1.91	95.25	0.00	2.63	0.00	0.00	2.12	95.23	0.00	2.62	0.00	0.00	2.15	88.70	0.00	11.30	0.00
0.1	0.3	0	0.00	95.10	2.50	0.00	1.00	0.00	2.40	94.99	2.59	0.00	1.00	0.00	2.42	97.06	2.94	0.00	1.00
0.1	0.3	0.5	0.50	94.84	2.60	2.56	0.50	0.00	0.00	94.96	2.50	2.54	0.50	0.00	0.00	96.92	1.02	2.06	0.33
0.1	0.3	1	1.00	94.86	2.74	2.40	0.53	0.00	0.00	94.78	2.79	2.43	0.53	0.00	0.00	96.02	0.15	3.83	0.04
0.1	0.3	1.5	1.50	94.82	2.40	2.65	0.48	0.00	0.11	94.90	2.33	2.66	0.47	0.00	0.11	94.70	0.00	5.30	0.00
0.1	0.3	2	2.00	95.25	0.00	2.47	0.00	0.00	2.28	95.22	0.00	2.50	0.00	0.00	2.28	94.35	0.00	5.65	0.00
0.3	0.1	0	0.00	94.97	2.63	0.00	1.00	1.76	1.53	95.02	2.61	0.00	1.00	1.60	1.47	99.70	0.30	0.00	1.00
0.3	0.1	0.5	0.47	95.09	1.58	2.35	0.40	0.20	0.04	95.07	1.64	2.34	0.41	0.19	0.02	93.93	0.00	6.07	0.00
0.3	0.1	1	0.95	94.81	1.04	2.66	0.28	0.03	0.17	94.89	1.01	2.67	0.27	0.04	0.15	89.31	0.00	10.69	0.00
0.3	0.1	1.5	1.43	95.19	0.80	2.39	0.25	0.00	0.59	95.23	0.81	2.39	0.25	0.00	0.59	89.22	0.00	10.78	0.00
0.3	0.1	2	1.95	95.14	0.00	2.45	0.00	0.00	2.41	95.04	0.00	2.56	0.00	0.00	2.40	89.25	0.00	10.75	0.00
0.3	0.3	0	0.00	94.90	2.50	0.00	1.00	0.00	2.60	94.85	2.51	0.00	1.00	0.00	2.64	97.11	2.89	0.00	1.00
0.3	0.3	0.5	0.50	94.88	2.59	2.52	0.51	0.00	0.00	94.96	2.59	2.45	0.51	0.00	0.00	96.93	1.11	1.96	0.36
0.3	0.3	1	1.00	95.30	2.41	2.28	0.51	0.00	0.00	95.31	2.44	2.25	0.52	0.00	0.00	96.03	0.16	3.81	0.04
0.3	0.3	1.5	1.51	94.95	2.51	2.45	0.51	0.00	0.08	94.96	2.48	2.48	0.50	0.00	0.08	95.10	0.00	4.90	0.00
0.3	0.3	2	2.00	95.30	0.00	2.21	0.00	0.00	2.49	95.20	0.00	2.30	0.00	0.00	2.50	94.55	0.00	5.45	0.00

[a]DP and EP of the delta method are zero

Table S4 Estimated median of the point estimates of γ, CP (in %), ML (in %), MR (in %), Ratio $(\mathrm{ML}/(\mathrm{ML}+\mathrm{MR}))$, DP (in %) and EP (in %) of two-sided 95% CIs of γ for the LR, Fieller's and delta methods against γ, with $a = 0.1$ and 0.3, $p = 0.1$ and 0.3, $n = 2{,}000$ and $\rho = 0.05$ based on $10{,}000$ replicates

				LR						Fieller						Delta[a]			
a	p	γ	Median	CP	ML	MR	Ratio	DP	EP	CP	ML	MR	Ratio	DP	EP	CP	ML	MR	Ratio
0.1	0.1	0	0.00	94.89	2.42	0.00	1.00	0.81	2.37	94.85	2.43	0.00	1.00	0.77	2.40	99.43	0.57	0.00	1.00
0.1	0.1	0.5	0.49	95.00	2.30	2.42	0.49	0.01	0.00	95.12	2.30	2.40	0.49	0.01	0.00	94.08	0.00	5.92	0.00
0.1	0.1	1	0.99	95.03	2.26	2.23	0.50	0.00	0.04	95.06	2.29	2.23	0.51	0.00	0.05	92.03	0.00	7.97	0.00
0.1	0.1	1.5	1.49	95.04	1.74	2.65	0.40	0.00	0.46	95.09	1.72	2.61	0.40	0.00	0.48	91.12	0.00	8.88	0.00
0.1	0.1	2	2.00	95.09	0.00	2.41	0.00	0.00	2.50	95.20	0.00	2.31	0.00	0.00	2.49	91.83	0.00	8.17	0.00
0.1	0.3	0	0.00	95.18	2.18	0.00	1.00	0.00	2.64	95.16	2.25	0.00	1.00	0.00	2.59	97.35	2.65	0.00	1.00
0.1	0.3	0.5	0.50	94.85	2.58	2.57	0.50	0.00	0.00	94.81	2.58	2.61	0.50	0.00	0.00	95.81	1.55	2.64	0.37
0.1	0.3	1	1.00	94.93	2.57	2.50	0.51	0.00	0.00	94.96	2.59	2.45	0.51	0.00	0.00	95.66	0.82	3.52	0.19
0.1	0.3	1.5	1.50	94.94	2.47	2.58	0.49	0.00	0.01	94.97	2.50	2.52	0.50	0.00	0.01	95.47	0.00	4.53	0.00
0.1	0.3	2	2.00	94.86	0.00	2.59	0.00	0.00	2.55	94.93	0.00	2.53	0.00	0.00	2.54	95.20	0.00	4.80	0.00
0.3	0.1	0	0.00	95.15	2.26	0.00	1.00	0.89	2.30	95.27	2.20	0.00	1.00	0.99	2.27	99.47	0.53	0.00	1.00
0.3	0.1	0.5	0.49	95.14	2.12	2.51	0.46	0.01	0.00	95.16	2.10	2.52	0.45	0.02	0.00	93.96	0.00	6.04	0.00
0.3	0.1	1	0.99	94.90	2.18	2.59	0.46	0.00	0.03	94.89	2.18	2.59	0.46	0.00	0.03	91.40	0.00	8.60	0.00
0.3	0.1	1.5	1.50	94.88	2.02	2.42	0.45	0.00	0.48	94.84	2.05	2.44	0.46	0.00	0.46	91.29	0.00	8.71	0.00
0.3	0.1	2	1.98	95.25	0.00	2.46	0.00	0.00	2.29	95.20	0.00	2.48	0.00	0.00	2.32	91.03	0.00	8.97	0.00
0.3	0.3	0	0.00	94.93	2.50	0.00	1.00	0.00	2.57	95.02	2.47	0.00	1.00	0.00	2.51	97.14	2.86	0.00	1.00
0.3	0.3	0.5	0.50	95.15	2.25	2.58	0.47	0.00	0.00	95.06	2.31	2.63	0.47	0.00	0.00	95.74	1.57	2.69	0.37
0.3	0.3	1	1.00	94.96	2.58	2.46	0.51	0.00	0.00	94.96	2.60	2.44	0.52	0.00	0.00	95.47	0.74	3.79	0.16
0.3	0.3	1.5	1.50	94.98	2.50	2.52	0.50	0.00	0.00	95.04	2.46	2.50	0.50	0.00	0.00	95.55	0.00	4.45	0.00
0.3	0.3	2	2.00	94.89	0.00	2.53	0.00	0.00	2.58	94.91	0.00	2.53	0.00	0.00	2.56	95.43	0.00	4.57	0.00

[a]DP and EP of the delta method are zero

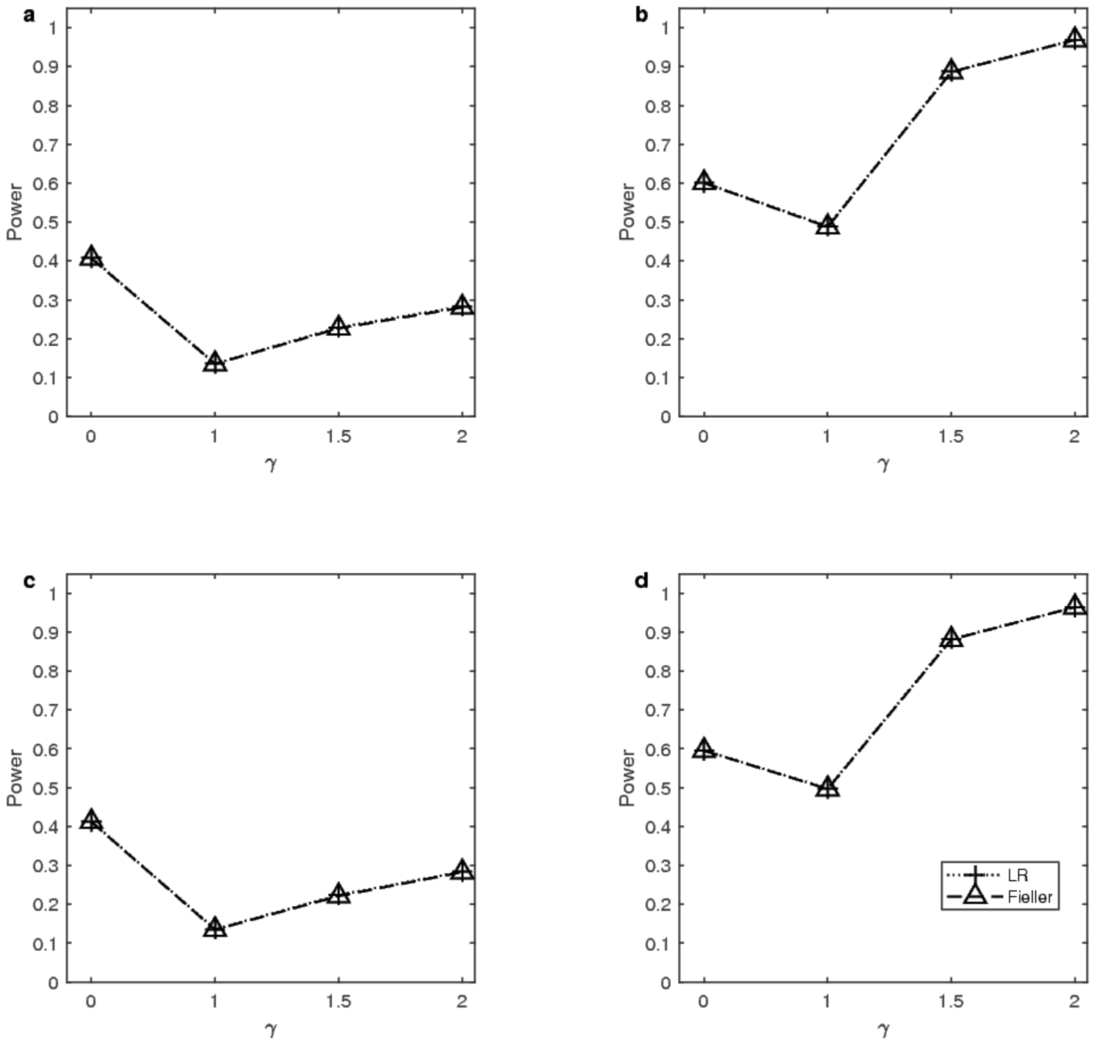

Fig. S1 Estimated powers for the LR and Fieller's methods against γ. The simulation is based on 10,000 replicates and 5% significance level with $n = 1{,}000$, $\rho = 0$ and $\gamma_0 = 0.5$. **a** $a = 0.1$, $p = 0.1$; **b** $a = 0.1$, $p = 0.3$; **c** $a = 0.3$, $p = 0.1$; **d** $a = 0.3$, $p = 0.3$

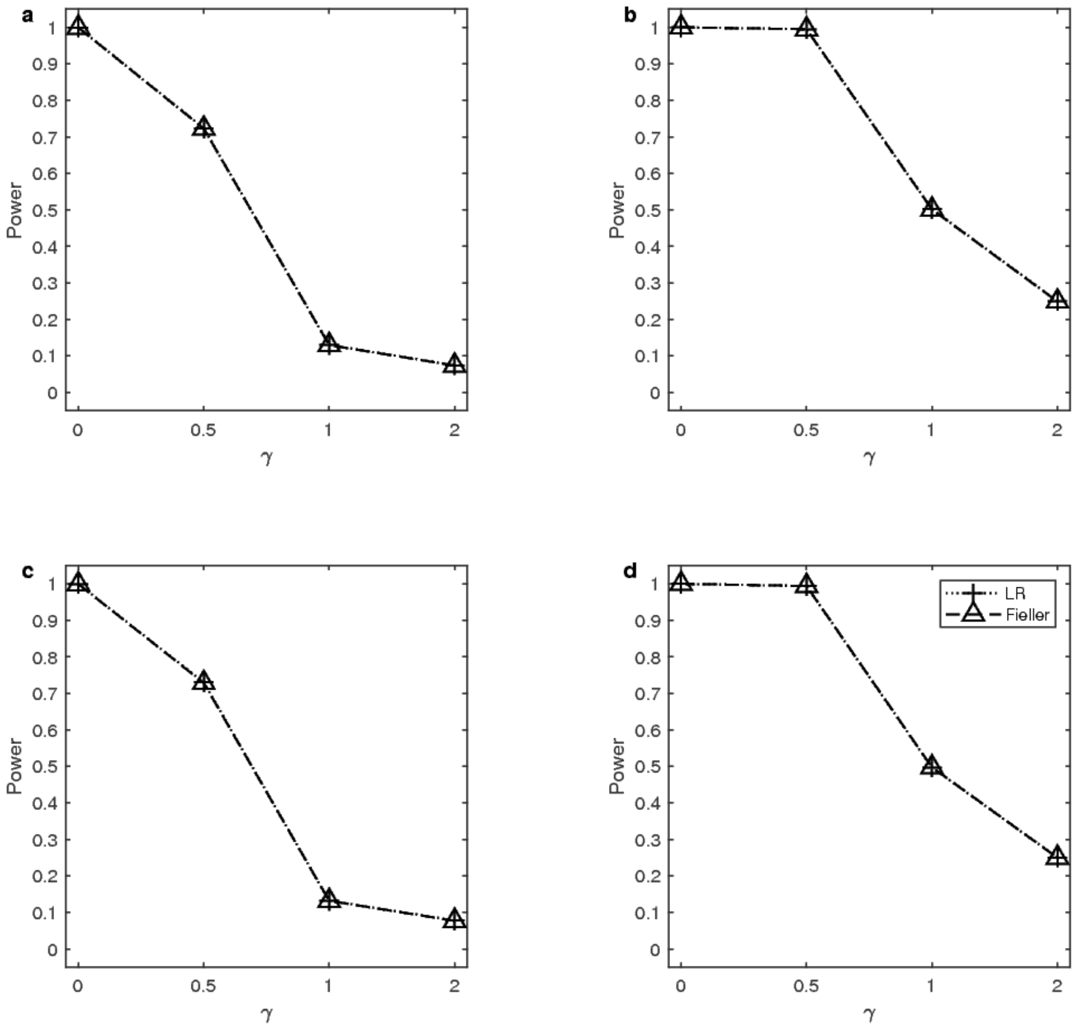

Fig. S2 Estimated powers for the LR and Fieller's methods against γ. The simulation is based on 10,000 replicates and 5% significance level with $n = 1{,}000$, $\rho = 0$ and $\gamma_0 = 1.5$. **a** $a = 0.1$, $p = 0.1$; **b** $a = 0.1$, $p = 0.3$; **c** $a = 0.3$, $p = 0.1$; **d** $a = 0.3$, $p = 0.3$

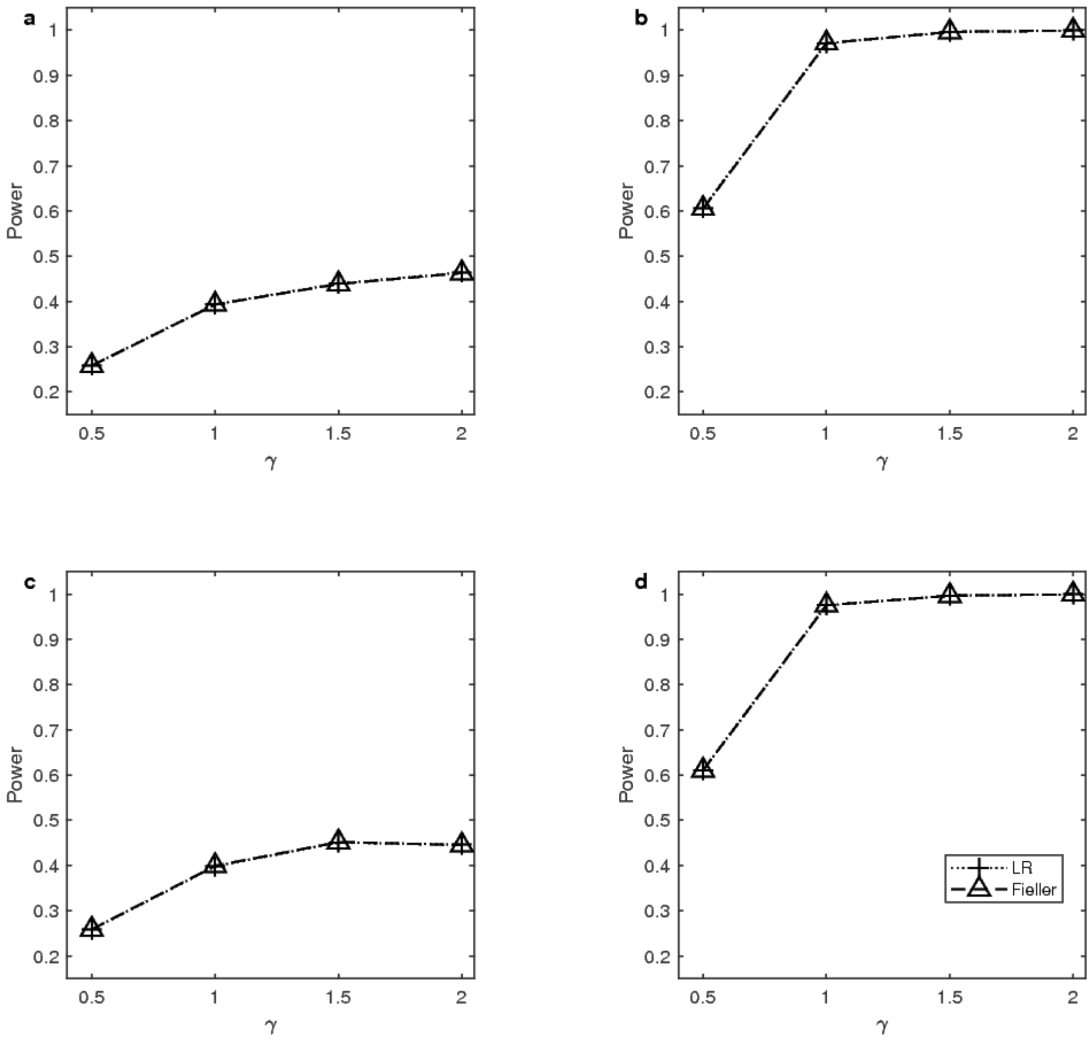

Fig. S3 Estimated powers for the LR and Fieller's methods against γ. The simulation is based on 10,000 replicates and 5% significance level with $n = 1{,}000$, $\rho = 0.05$ and $\gamma_0 = 0$. **a** $a = 0.1$, $p = 0.1$; **b** $a = 0.1$, $p = 0.3$; **c** $a = 0.3$, $p = 0.1$; **d** $a = 0.3$, $p = 0.3$

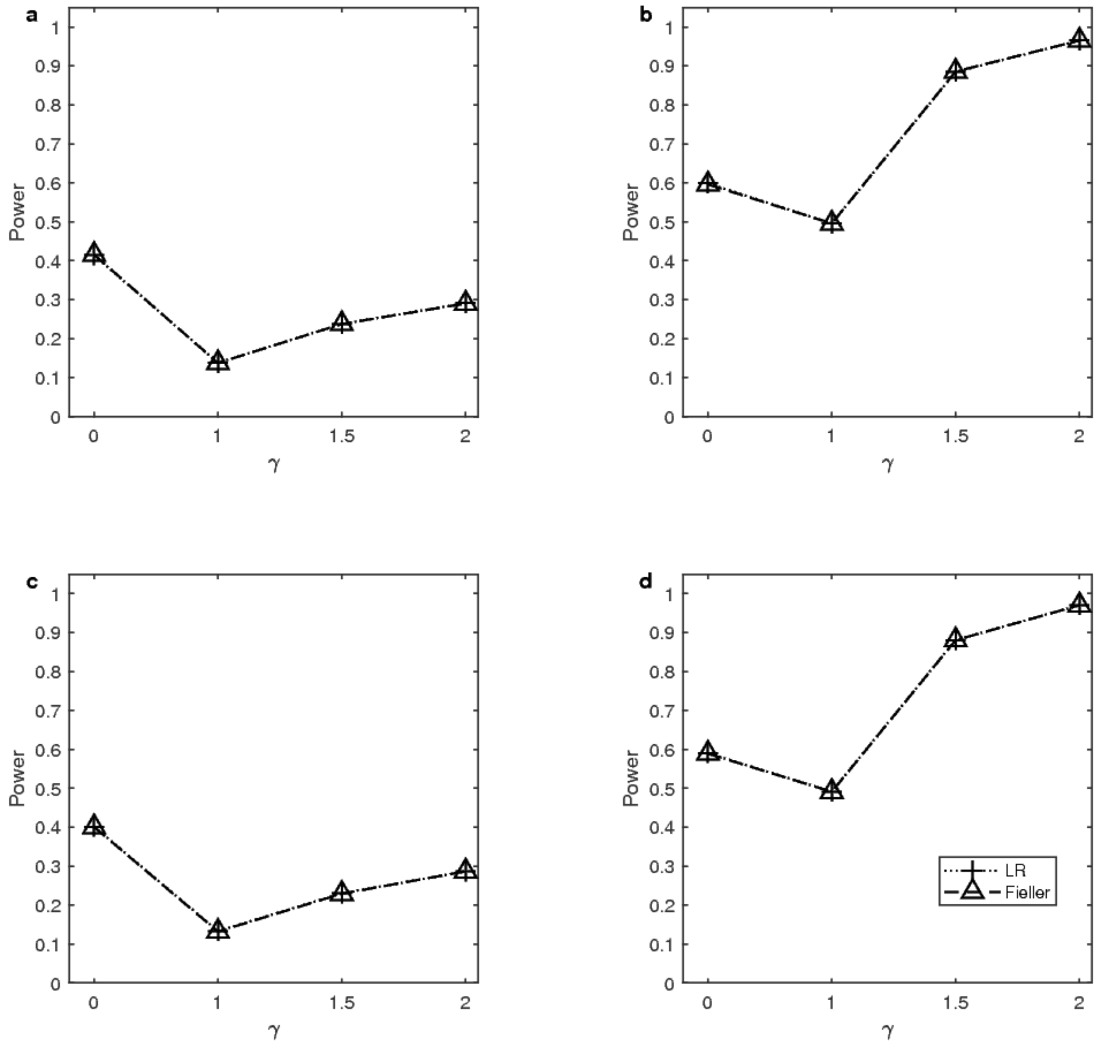

Fig. S4 Estimated powers for the LR and Fieller's methods against γ. The simulation is based on 10,000 replicates and 5% significance level with $n = 1{,}000$, $\rho = 0.05$ and $\gamma_0 = 0.5$. **a** $a = 0.1,\ p = 0.1$; **b** $a = 0.1,\ p = 0.3$; **c** $a = 0.3,\ p = 0.1$; **d** $a = 0.3,\ p = 0.3$

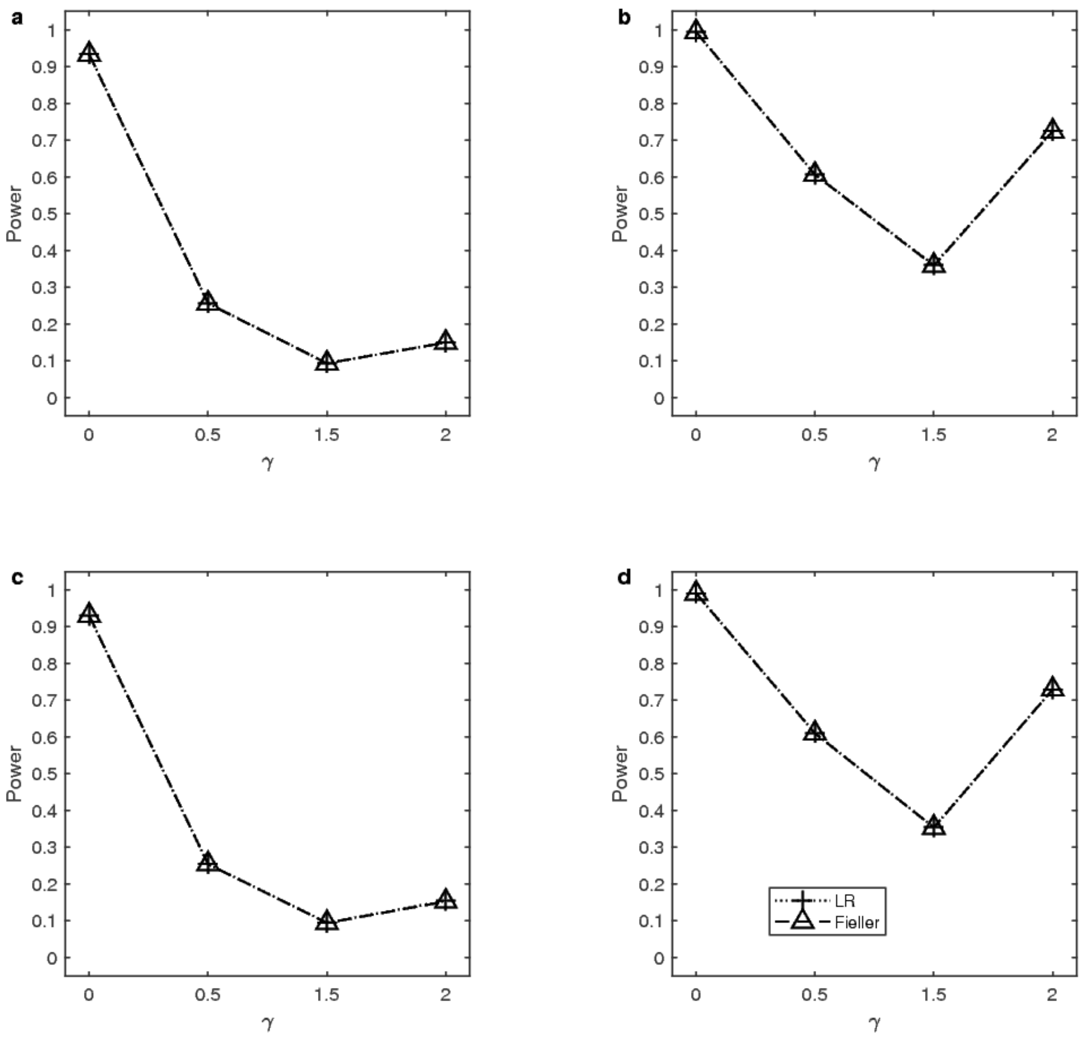

Fig. S5 Estimated powers for the LR and Fieller's methods against γ. The simulation is based on 10,000 replicates and 5% significance level with $n = 1{,}000$, $\rho = 0.05$ and $\gamma_0 = 1$. **a** $a = 0.1$, $p = 0.1$; **b** $a = 0.1$, $p = 0.3$; **c** $a = 0.3$, $p = 0.1$; **d** $a = 0.3$, $p = 0.3$

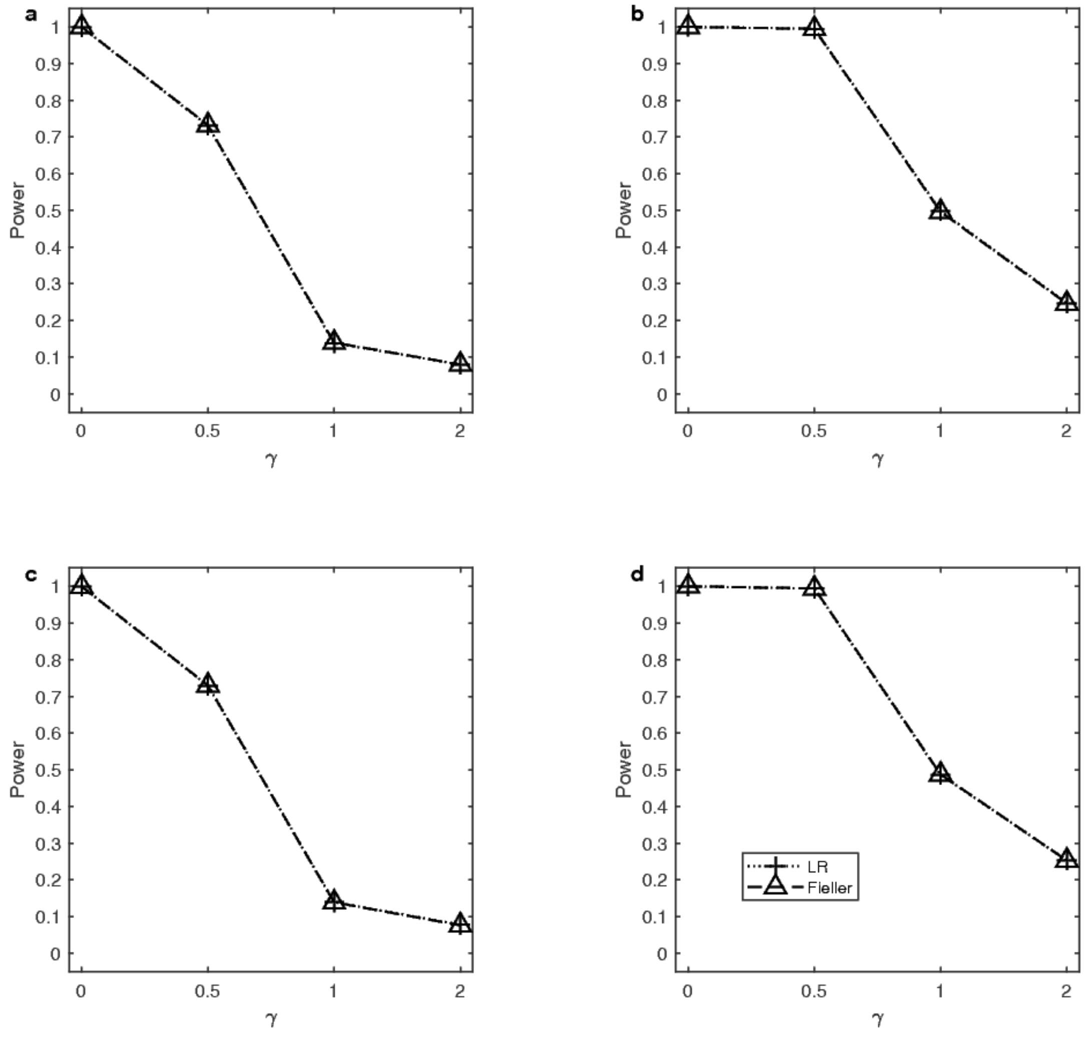

Fig. S6 Estimated powers for the LR and Fieller's methods against γ. The simulation is based on 10,000 replicates and 5% significance level with $n = 1{,}000$, $\rho = 0.05$ and $\gamma_0 = 1.5$. **a** $a = 0.1$, $p = 0.1$; **b** $a = 0.1$, $p = 0.3$; **c** $a = 0.3$, $p = 0.1$; **d** $a = 0.3$, $p = 0.3$

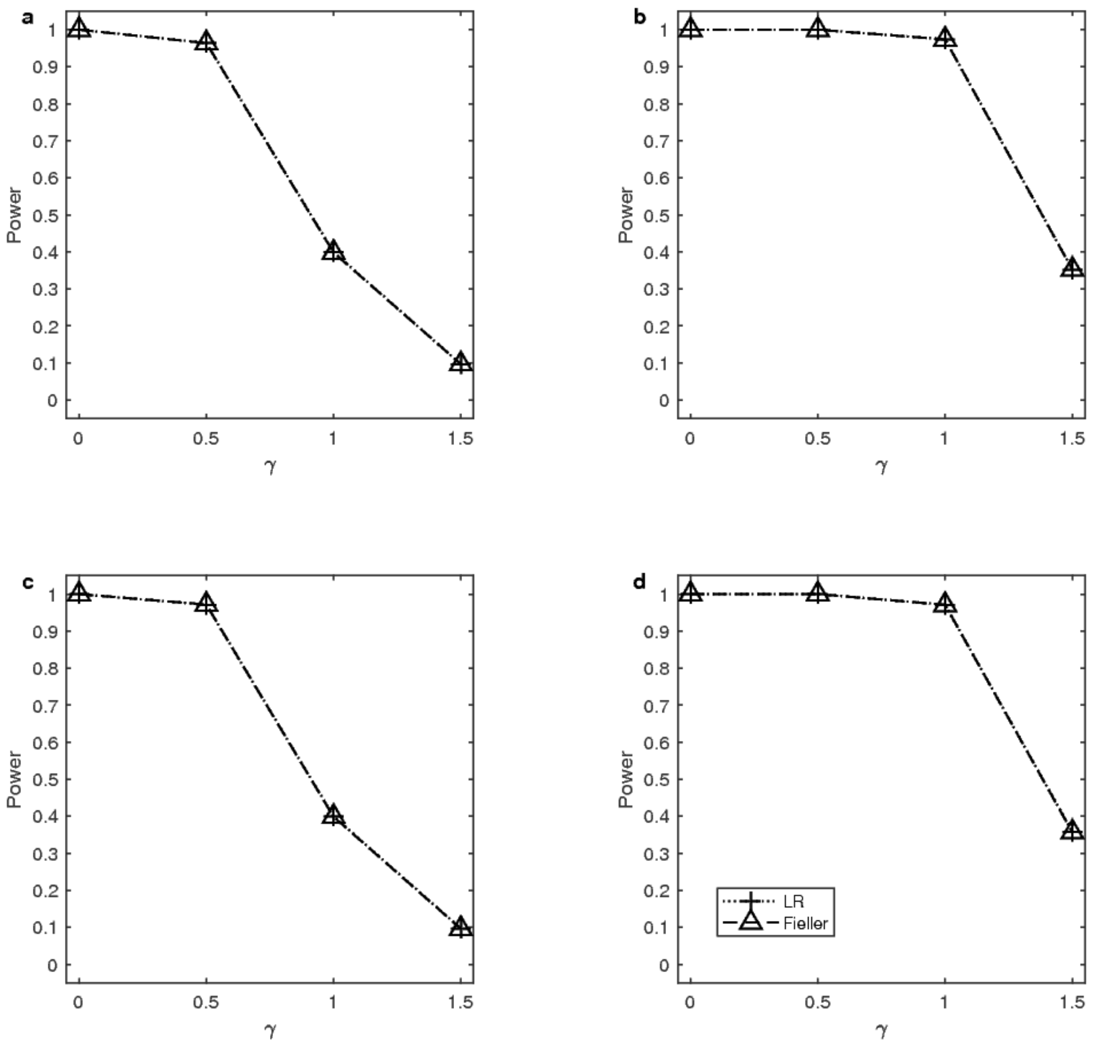

Fig. S7 Estimated powers for the LR and Fieller's methods against γ. The simulation is based on 10,000 replicates and 5% significance level with $n = 1{,}000$, $\rho = 0.05$ and $\gamma_0 = 2$. **a** $a = 0.1$, $p = 0.1$; **b** $a = 0.1$, $p = 0.3$; **c** $a = 0.3$, $p = 0.1$; **d** $a = 0.3$, $p = 0.3$

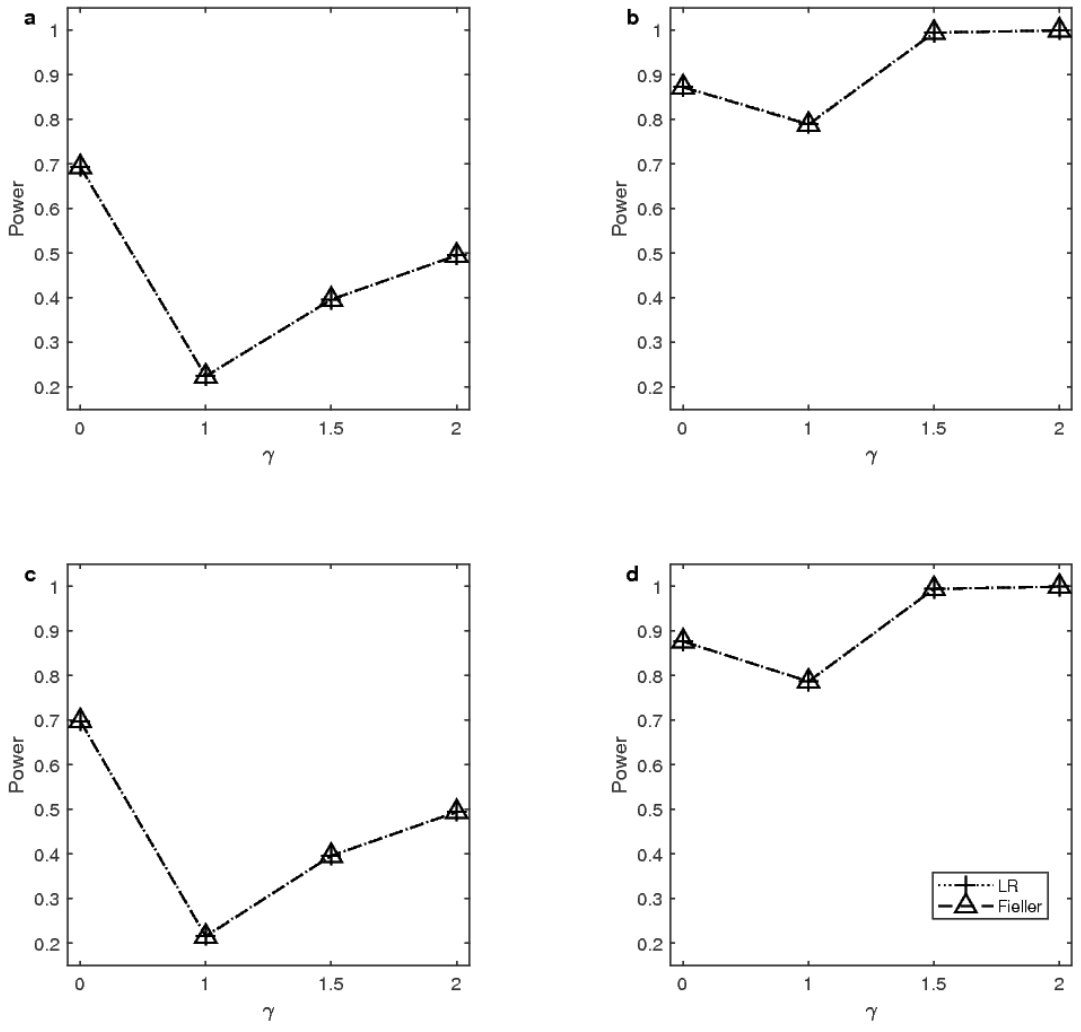

Fig. S8 Estimated powers for the LR and Fieller's methods against γ. The simulation is based on 10,000 replicates and 5% significance level with $n = 2{,}000$, $\rho = 0$ and $\gamma_0 = 0.5$. **a** $a = 0.1$, $p = 0.1$; **b** $a = 0.1$, $p = 0.3$; **c** $a = 0.3$, $p = 0.1$; **d** $a = 0.3$, $p = 0.3$

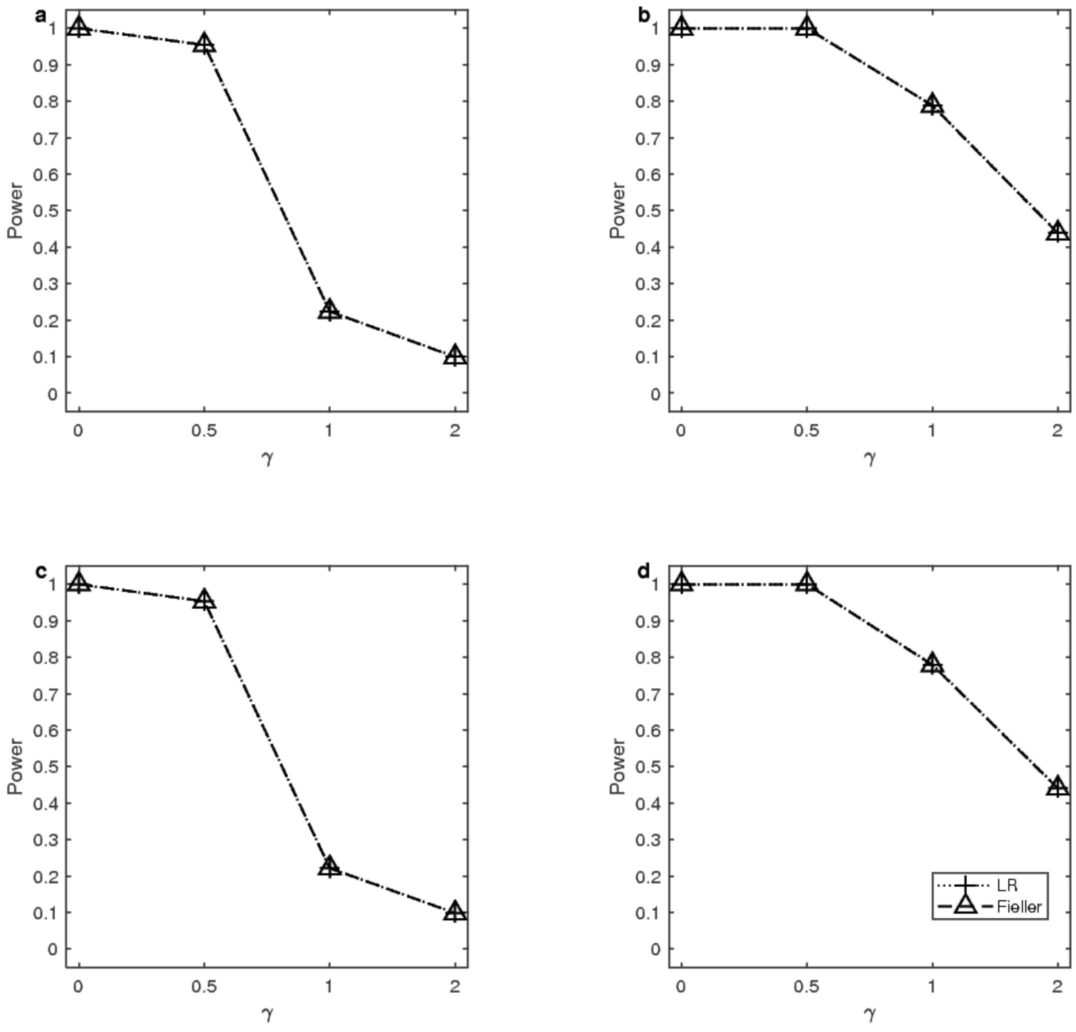

Fig. S9 Estimated powers for the LR and Fieller's methods against γ. The simulation is based on 10,000 replicates and 5% significance level with $n = 2{,}000$, $\rho = 0$ and $\gamma_0 = 1.5$. **a** $a = 0.1$, $p = 0.1$; **b** $a = 0.1$, $p = 0.3$; **c** $a = 0.3$, $p = 0.1$; **d** $a = 0.3$, $p = 0.3$

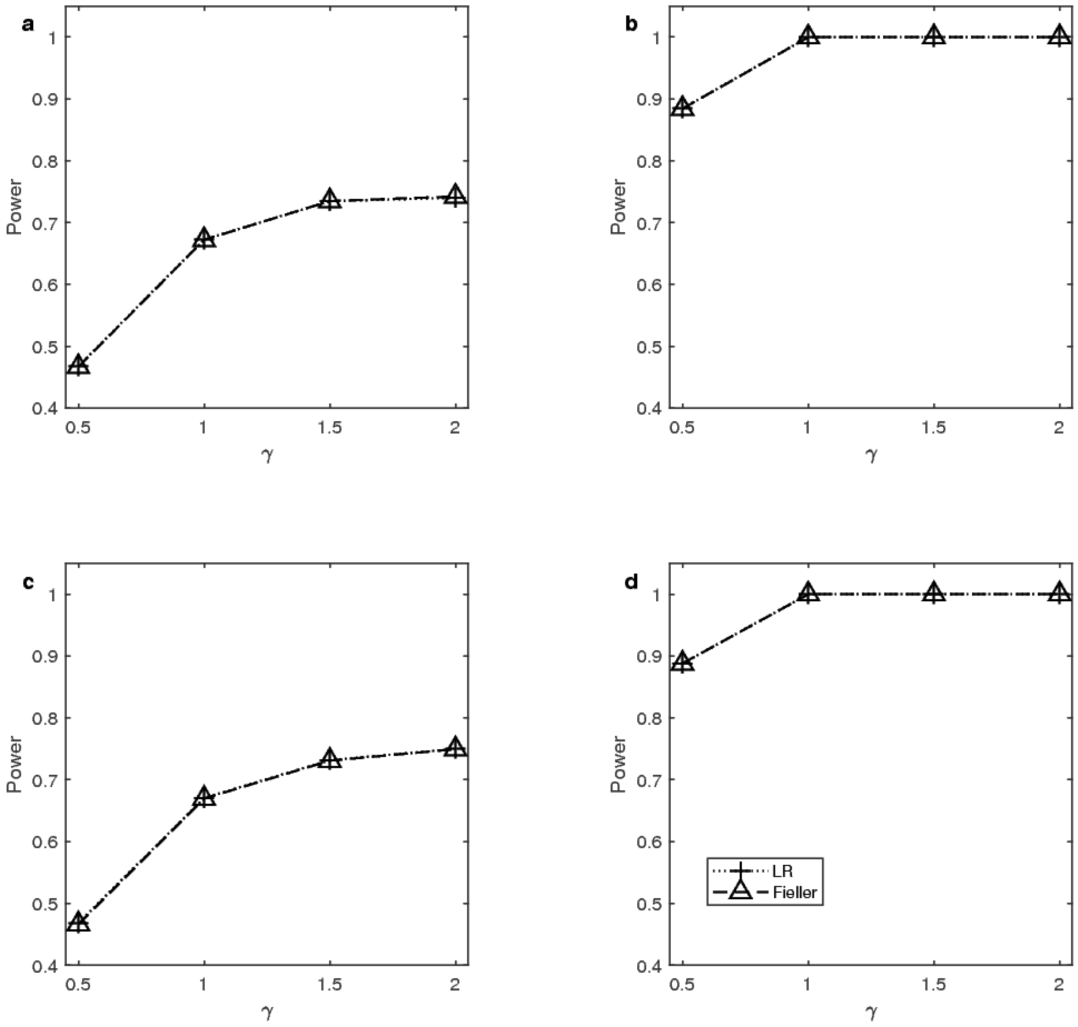

Fig. S10 Estimated powers for the LR and Fieller's methods against γ. The simulation is based on 10,000 replicates and 5% significance level with $n = 2{,}000$, $\rho = 0.05$ and $\gamma_0 = 0$. **a** $a = 0.1$, $p = 0.1$; **b** $a = 0.1$, $p = 0.3$; **c** $a = 0.3$, $p = 0.1$; **d** $a = 0.3$, $p = 0.3$

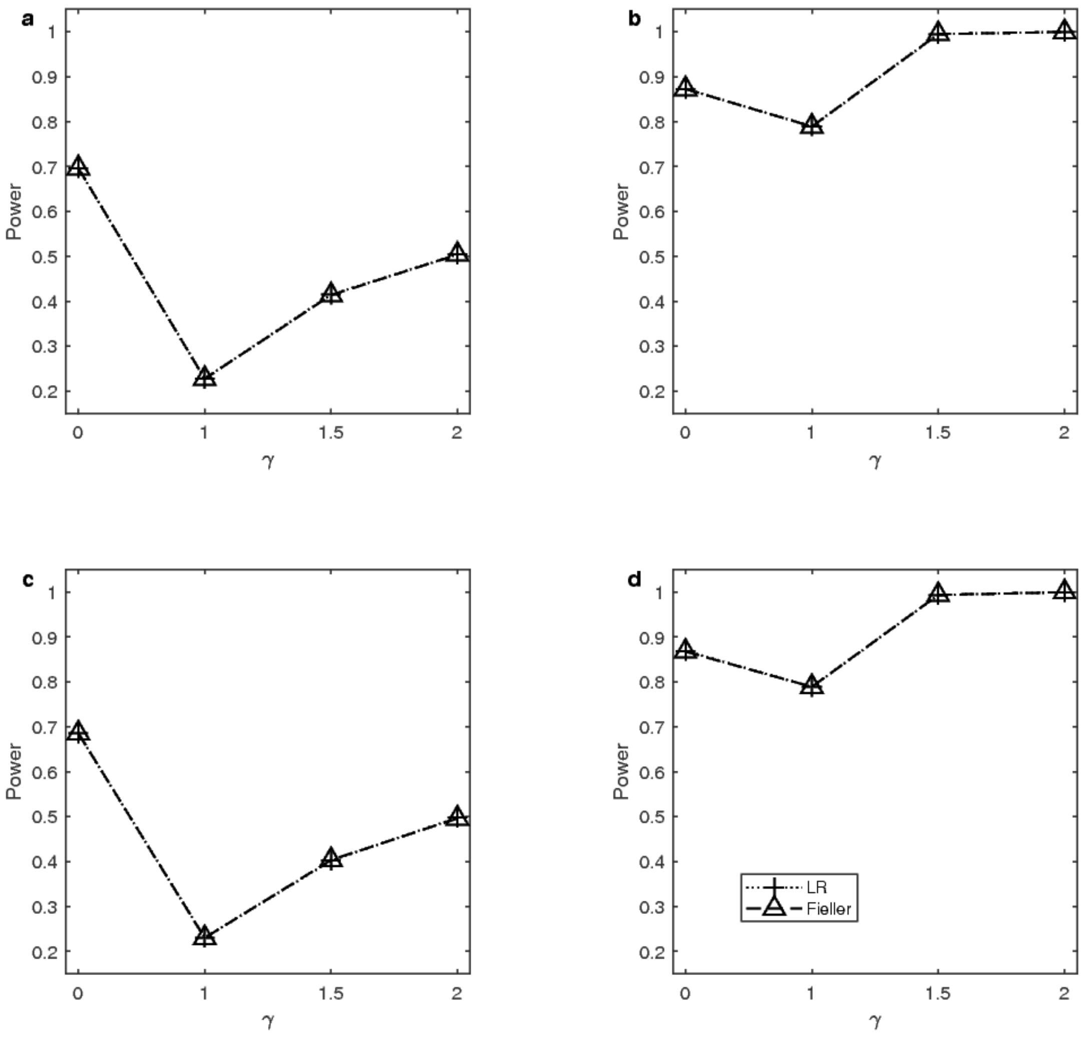

Fig. S11 Estimated powers for the LR and Fieller's methods against γ. The simulation is based on 10,000 replicates and 5% significance level with $n = 2{,}000$, $\rho = 0.05$ and $\gamma_0 = 0.5$. **a** $a = 0.1$, $p = 0.1$; **b** $a = 0.1$, $p = 0.3$; **c** $a = 0.3$, $p = 0.1$; **d** $a = 0.3$, $p = 0.3$

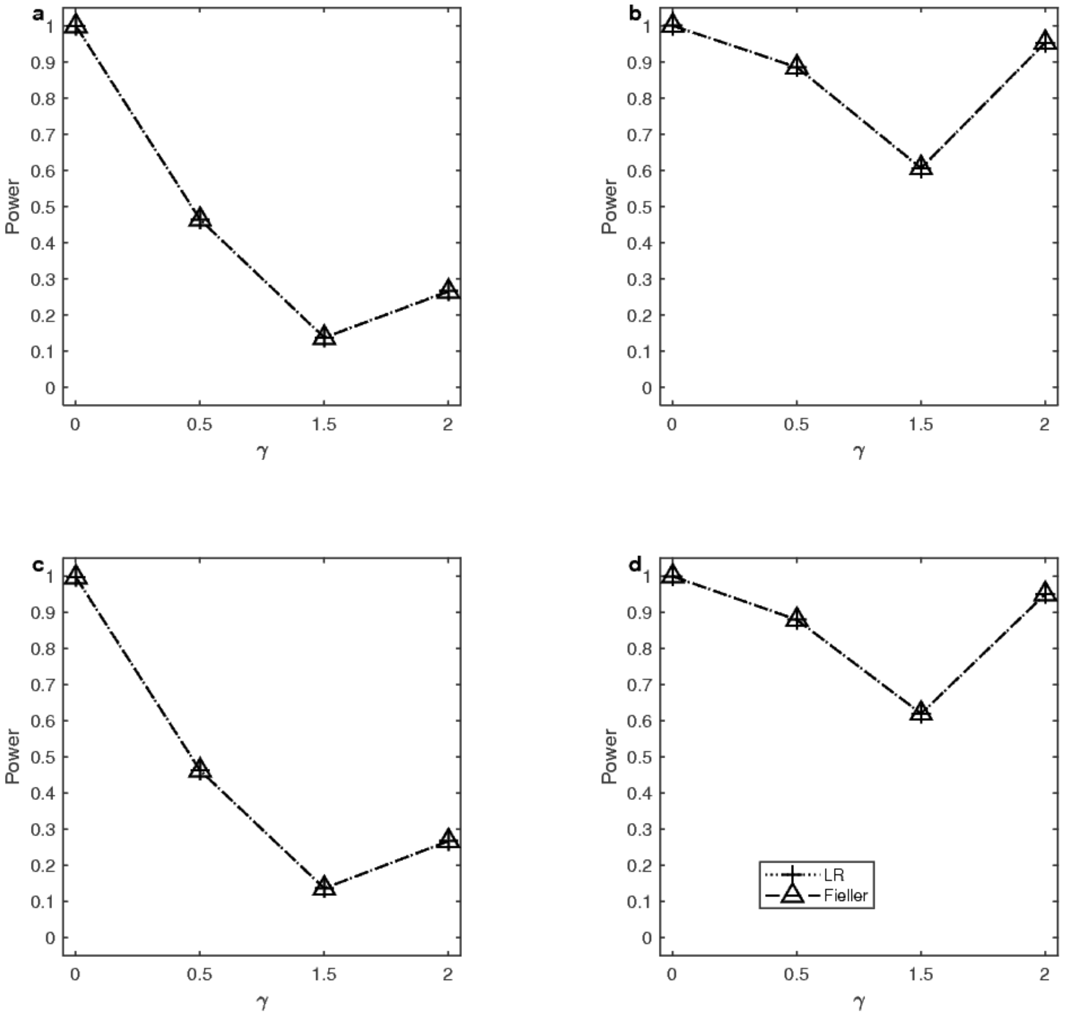

Fig. S12 Estimated powers for the LR and Fieller's methods against γ. The simulation is based on 10,000 replicates and 5% significance level with $n = 2{,}000$, $\rho = 0.05$ and $\gamma_0 = 1$. **a** $a = 0.1$, $p = 0.1$; **b** $a = 0.1$, $p = 0.3$; **c** $a = 0.3$, $p = 0.1$; **d** $a = 0.3$, $p = 0.3$

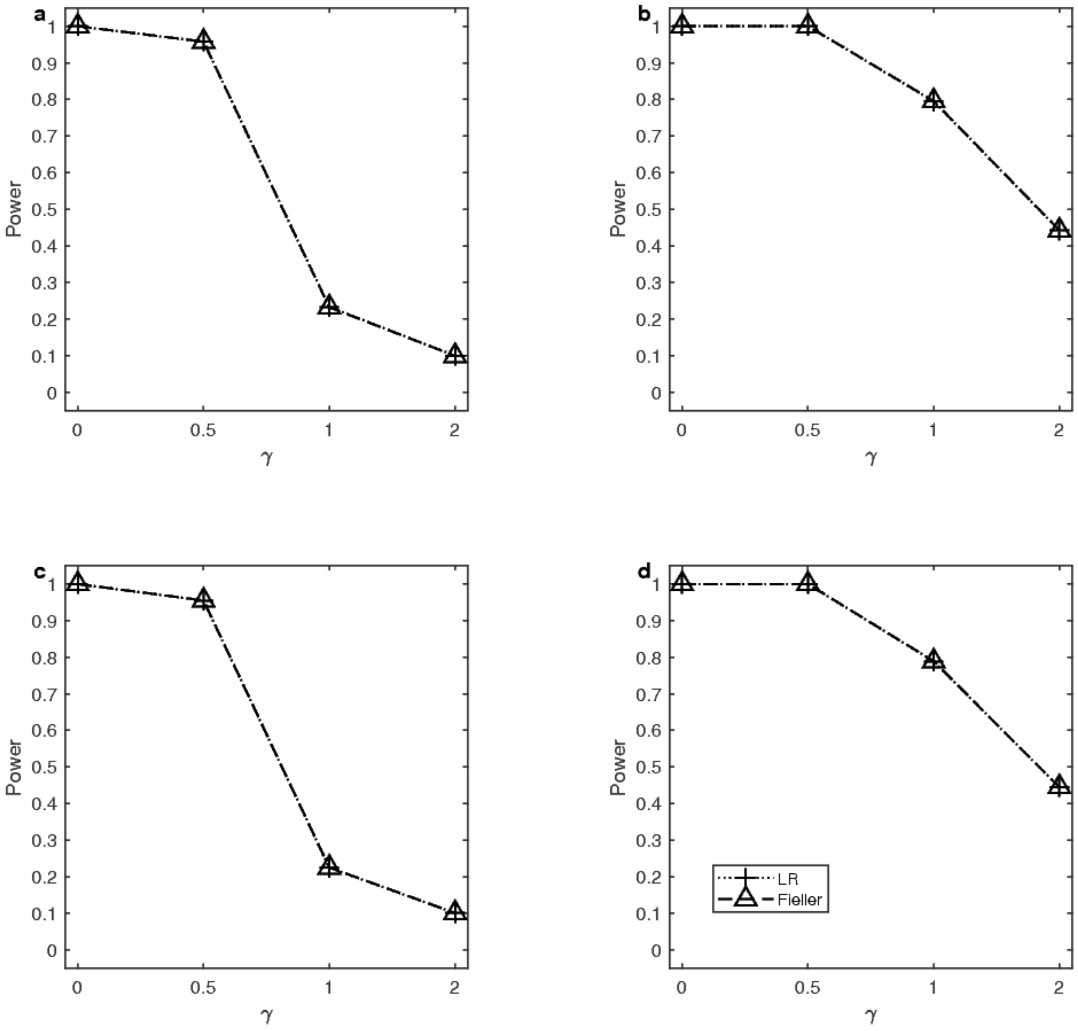

Fig. S13 Estimated powers for the LR and Fieller's methods against γ. The simulation is based on 10,000 replicates and 5% significance level with $n = 2{,}000$, $\rho = 0.05$ and $\gamma_0 = 1.5$. **a** $a = 0.1$, $p = 0.1$; **b** $a = 0.1$, $p = 0.3$; **c** $a = 0.3$, $p = 0.1$; **d** $a = 0.3$, $p = 0.3$

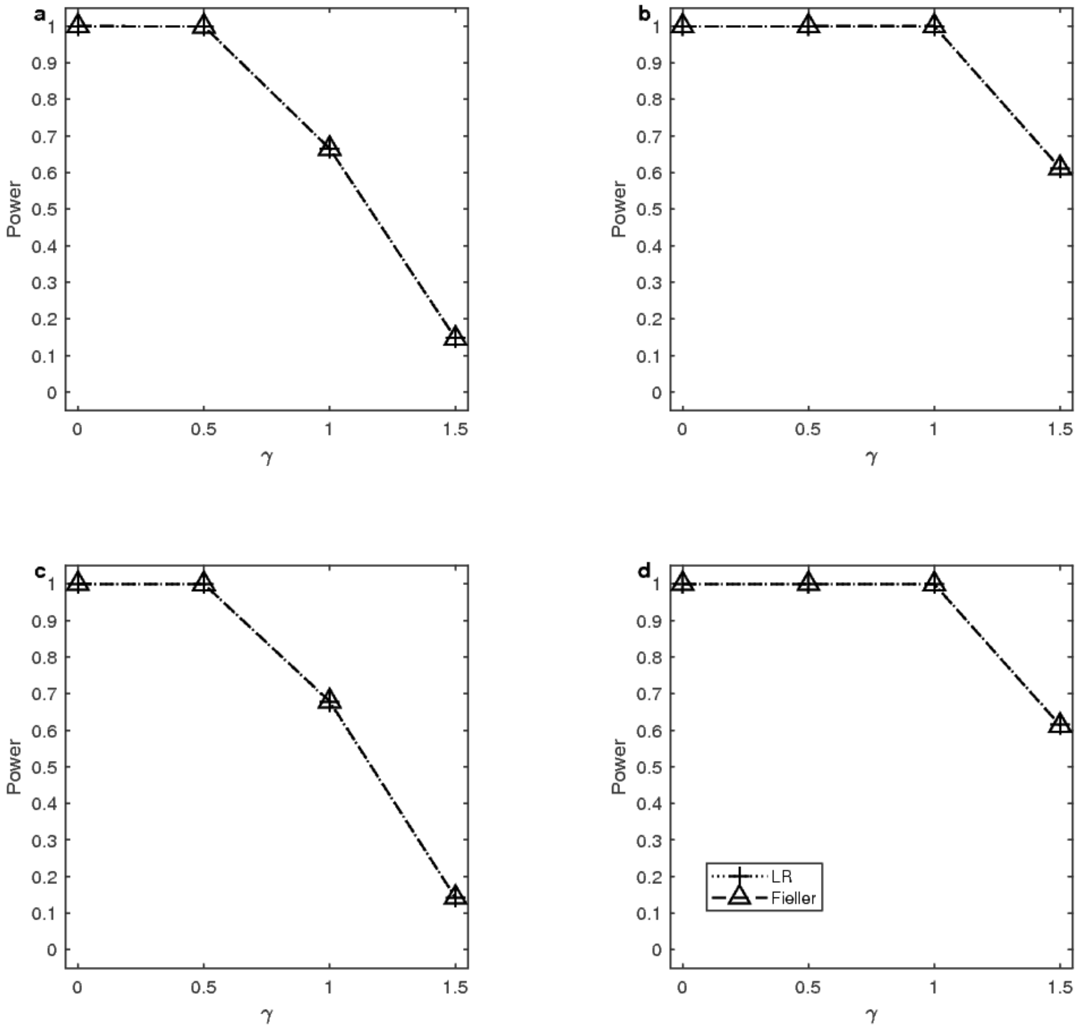

Fig. S14 Estimated powers for the LR and Fieller's methods against γ. The simulation is based on 10,000 replicates and 5% significance level with $n = 2{,}000$, $\rho = 0.05$ and $\gamma_0 = 2$. **a** $a = 0.1$, $p = 0.1$; **b** $a = 0.1$, $p = 0.3$; **c** $a = 0.3$, $p = 0.1$; **d** $a = 0.3$, $p = 0.3$
